# Resurgence and Repurposing of Antifungal Azoles by Transition Metal Coordination for Drug Discovery

**DOI:** 10.3390/pharmaceutics15102398

**Published:** 2023-09-28

**Authors:** Youri Cortat, Fabio Zobi

**Affiliations:** Department of Chemistry, University of Fribourg, Chemin du Musée 9, 1700 Fribourg, Switzerland; youri.cortat@unifr.ch

**Keywords:** repurposing, antifungal, azole, metal, complexes, miconazole, clotrimazole, ketoconazole, fluconazole

## Abstract

Coordination compounds featuring one or more antifungal azole (AA) ligands constitute an interesting family of candidate molecules, given their medicinal polyvalence and the viability of drug complexation as a strategy to improve and repurpose available medications. This review reports the work performed in the field of coordination derivatives of AAs synthesized for medical purposes by discussing the corresponding publications and emphasizing the most promising compounds discovered so far. The resulting overview highlights the efficiency of AAs and their metallic species, as well as the potential still lying in this research area.

## 1. Introduction

The lack of new medically active molecules remains an issue for the treatment of several diseases and infections. In particular, cancer is responsible for approximately one death in six worldwide, and its incidence will probably raise from 18.1 million cases in 2018 to 29.4 million in 2040, according to the World Health Organization report on cancer of 2020 [1]. At the same time, antimicrobial resistance (AMR) poses a serious threat to global health, possibly causing 10 million death a year by 2050 if no efficient measures are rapidly taken [2]. Indeed, the last class of antibiotics was discovered more than 30 years ago. Since then, big pharmaceutical companies have scarcely invested in the development of new antibiotics due to an absence of financial interest [3]. Moreover, pathogens of the bacteria, fungi and protozoa groups known for developing AMR are responsible for certain neglected tropical diseases (NTDs), which are causing devastating human and material consequences in developing countries. Namely, NTDs are estimated to affect 1.7 billion people, causing 200,000 deaths each year [4].

**Scheme 1:**
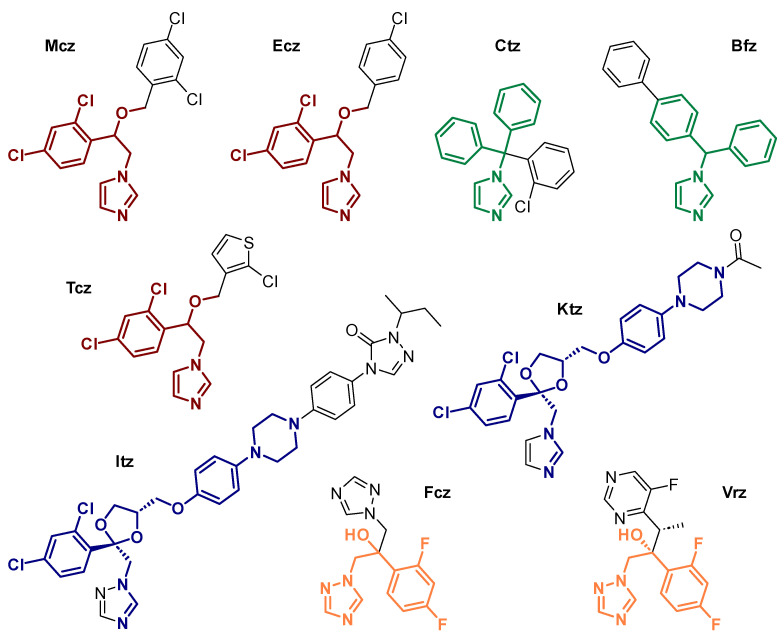
Structure of selected examples of antifungal azoles (AAs): miconazole (Mcz), econazole (Ecz), tioconazole (Tcz), clotrimazole (Ctz), bifonazole (Bfz), ketoconazole (Ktz), itraconazole (Itz), fluconazole (Fcz) and voriconazole (Vrz), with emphasized structural similarities within the same family of AAs.

**Figure 1:**
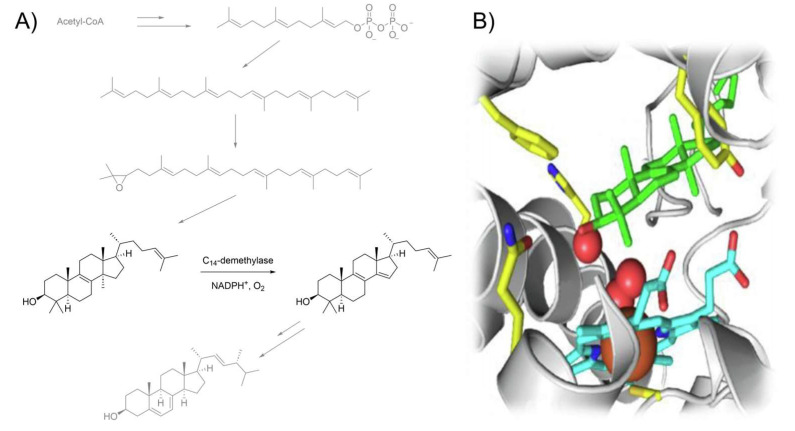
(**A**) Fungal ergosterol biosynthesis with lanosterol demethylation highlighted. (**B**) Schematic diagram of CYP51 catalytic site: lanosterol (green), heme (cyan), iron (orange); obtained and modified with permission [11].

Antifungal azoles (AAs, Scheme 1) form a class of therapeutic compounds that display selective activity against fungal pathogens by blocking ergosterol biosynthesis through the inhibition of lanosterol C_14_-demethylation (Figure 1A). This process occurs via coordination to the iron heme porphyrin moiety of the CYP51 enzyme (Figure 1B), which makes it impossible to perform an oxidative removal of the lanosterol methyl group [5,6,7]. As a consequence, the fungal organism experiences sterol depletion and accumulation of derivatives that are unable to orientate correctly within the phospholipid bilayer, ultimately leading to loss of membrane integrity and cell lysis [8,9]. Although concomitant binding to human enzymes can cause serious side effects [10], careful tuning of the drug structure and recognition of the medically relevant moieties through structure–activity relationship (SAR) studies have enabled the design of more viable generations of AAs. The resulting selectivity is quite remarkable, considering the higher similarity between humans and fungi compared to prokaryotic parasites [11].

Limiting AAs to their fungicidal properties would be shortsighted. Indeed, their ability to interact with CYP51 allows the inhibition of sterol biosynthesis in other organisms as well. In particular, activity against *T. cruzi*, the protozoan parasite causing the Chaga’s disease, and certain *Leshmania* species were reported, as these organisms share a similar ergosterol pathway compared to fungi [12,13].

**Figure 2:**
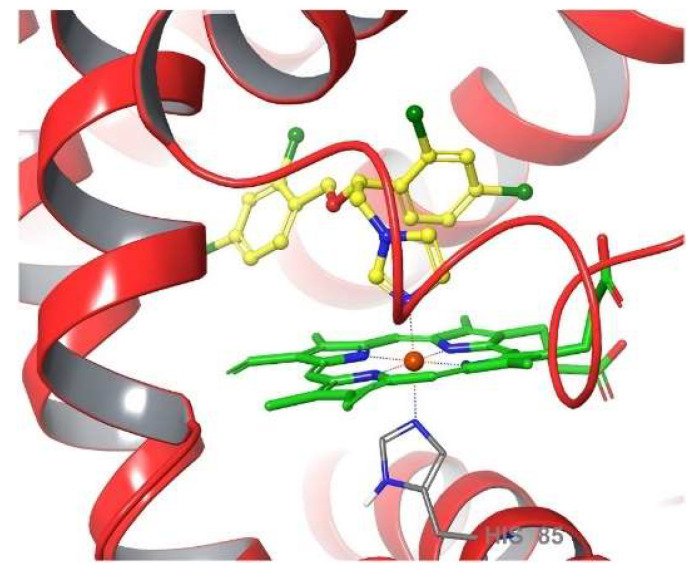
Schematic diagram of *R. eutropha* flavohemoglobin catalytic site with bound miconazole shown in yellow; obtained and modified with permission [17].

AAs have shown their potential against prokaryotic pathogens too, as they have been known for a few decades to exhibit antibacterial activity [14,15]. In this case, the drug mechanism slightly differs from the one observed in fungal and protozoan species. Indeed, the AA probably binds the iron heme porphyrin of a bacterial deoxygenase called flavohemoglobin (Figure 2), preventing the conversion of nitrogen monoxide into nitrate by occupying the coordination site. The inability to expel NO molecules renders the bacterial organism vulnerable to NO-mediated damage induced, e.g., by host immune cells [16]. New evidence toward this mode of action was recently afforded by computational methods analyzing more than one hundred AAs and some of their close derivatives [17].

Finally, certain AAs have been studied for their anticancer properties, beginning with the investigation of ketoconazole (Ktz) as an androgen blocker through the inhibition of CYP17A1 enzyme for the treatment of prostate cancer [18]. Later, the cytotoxicity of miconazole (Mcz), econazole (Ecz), clotrimazole (Ctz) and itraconazole (Itz) was assessed in several publications, and proven to occur by various mechanisms of action including Ca^2+^ depletion, cell cycle arrest, glycolysis disturbance and the inhibition of the Hedgehog pathway [19]. The latter effect is especially recurrent in the case of Itz [20]. Other AAs, namely bifonazole (Bfz) [21] and tioconazole (Tcz) [22], have been scarcely analyzed for antitumoral purposes, while fluconazole (Fcz) and voriconazole (Vrz) yielded poor results in this medical application [23].

The potential combination of anticancer and antimicrobial activity in a single molecule obviously presents some advantages, considering, e.g., the necessity of prophylactic antifungal therapy in the case of immunocompromised patients who undergo certain transplants or chemotherapeutic treatments [24]. Infections caused by *Candida* sp. in hospitals have to be emphasized as well, because they remain a serious danger during prolonged antibiotic therapies, while multidrug treatments always carry the risk of undesired drug–drug interactions [25]. Furthermore, successfully extending the use of approved drugs outside of their original prescription constitutes a considerable gain of time and financial resources [26]. However, the repurposing of antimicrobial drugs as anticancer agents goes against the current measures preventing antibiotics misuse. It was indeed reported that such treatments promoted AMR in late stage cancer patients, threatening the health of other immunocompromised people [27].

Despite the above optimistic considerations about AAs, they are by no means new drugs and thus fail to answer the need for novel therapeutic agents. However, their Lewis basicity allows coordination to a metal center, as evidenced by their mechanisms of action. Metal complexation of well-established drugs constitutes a promising strategy to overcome loss of medication sensitivity and induced resistance [28]. Apart from the important biological activity of the metal ions, administrated in the form of approved metallodrugs [29,30,31,32,33], organometallic chemistry allows wider diversity in the design of medically relevant molecules, notably through higher number of possible geometries, redox features, non-covalent bonding and catalytic properties [29]. Moreover, the coordinated derivatives of biologically active ligands have been known to show synergistic effect with certain metal cores. This phenomenon, often referred to as ‘metal-drug synergism’ (MDS), can be explained by both an increase in drug activity, due to stabilization via complexation, leading to longer residence time, and a decrease in the metal toxicity, thanks to limited availability for undesired reactions compared, e.g., to the free hydrated ion [34]. In some cases, metal ions can regulate the activity of an organic drug or vice versa without requiring the intake of the corresponding metallodrug. Such examples concerning AAs are manifold in the literature [35,36,37,38,39,40,41,42,43,44,45,46,47].

The present review aims to report the investigations conducted into transition metal coordination compounds of AAs, in order to offer an overview of the research situation in this topic. Publications reporting the synthesis of metallic AA derivatives without discussing their medical relevance, and the ones addressing nanoparticles or other nanomaterials, are excluded here. In this contribution, the following sections are organized according to the structural similarities between certain AAs (Scheme 1), with the resulting families being treated separately. The Mcz family and all its related papers in the field of coordination chemistry are discussed first, followed by derivatives of Ctz, Ktz and Fcz.

## 2. Antimicrobial and Anticancer Activity of Transition Metal Coordination Compounds Bearing AAs

### 2.1. Coordination Compounds of the Mcz Family of AAs

Developed by Janssen in 1968, Mcz entered the market as a topical antifungal agent in 1971. As the first synthesized and approved medically relevant AA, Mcz marked the advent of the first generation of these compounds. Simultaneously, Ecz was patented by the same company and a few years later, Tcz was designed by Pfizer [48]. Structurally speaking, these AAs are closely related to one another, as they all feature a metal-coordinating imidazole (imz), a dichlorophenyl ring and an etheric oxygen linked by an ethyl scaffold (Scheme 1). They differ from each other by the ether group bearing an aryl- or heteroarylmethyl moiety. As the predecessor of all AAs, Mcz remains the most investigated one in the field of coordination chemistry. Surprisingly, Tcz holds second place, despite Ecz displaying almost the same structure as Mcz.

**Scheme 2:**
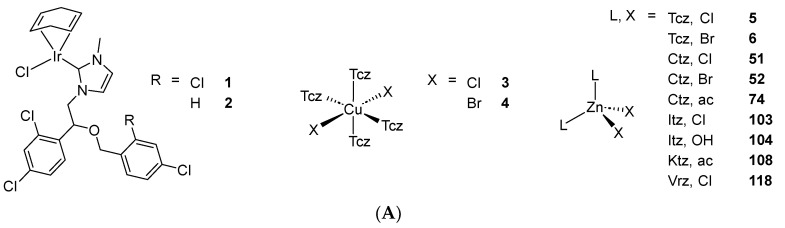

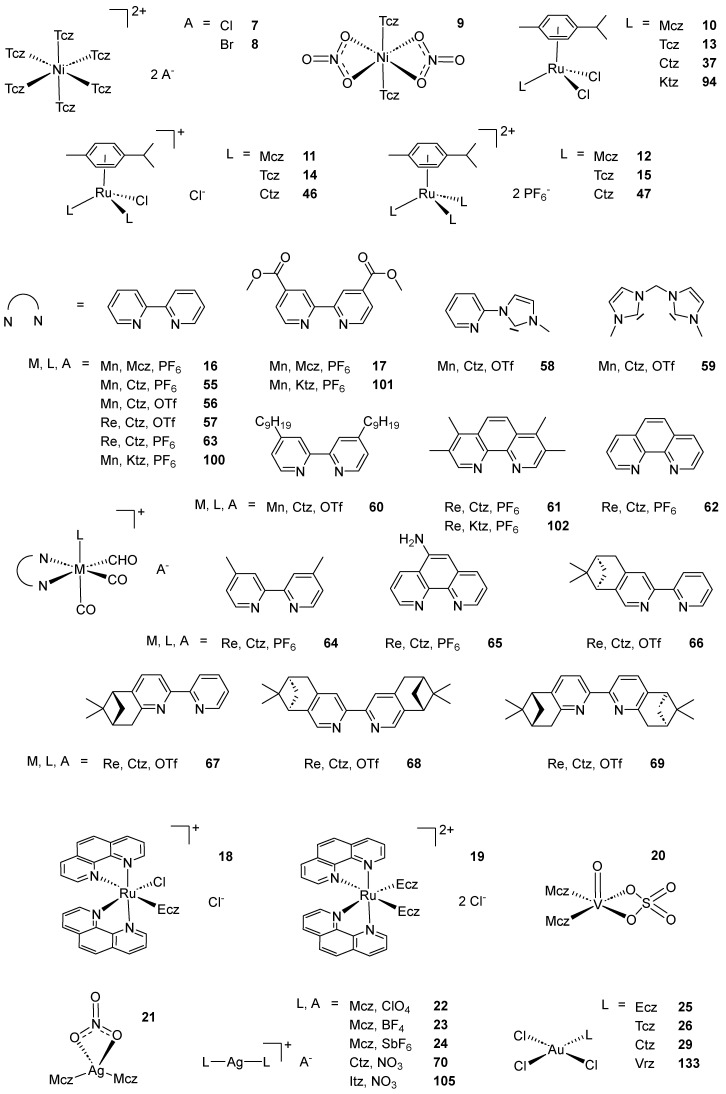

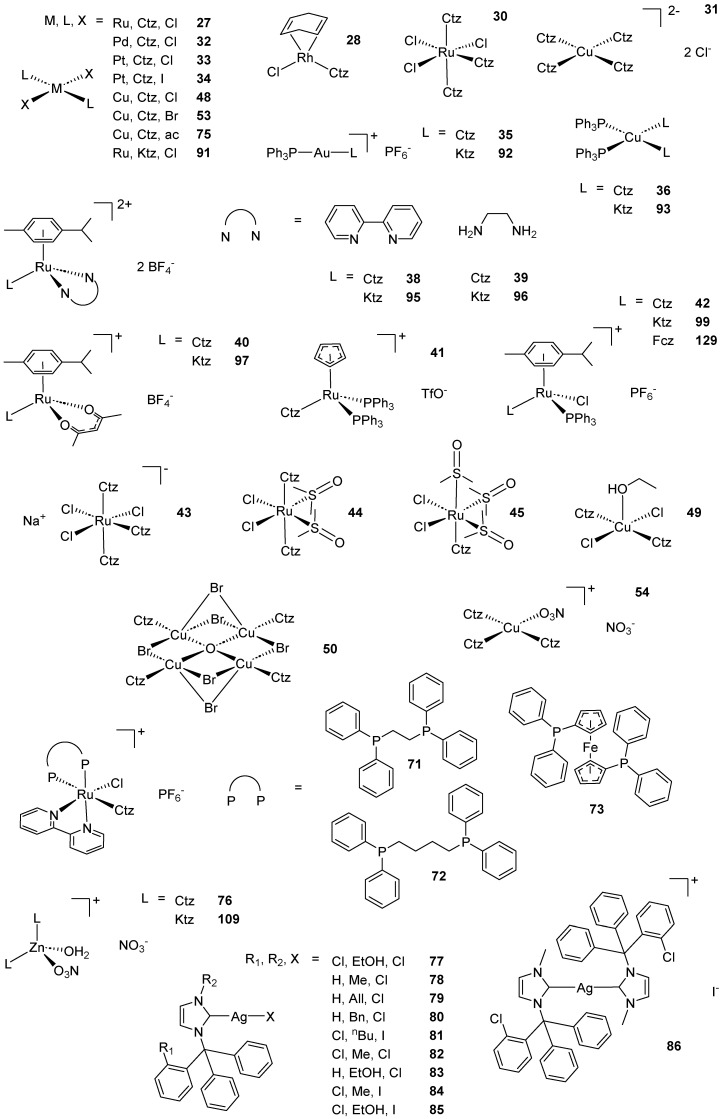

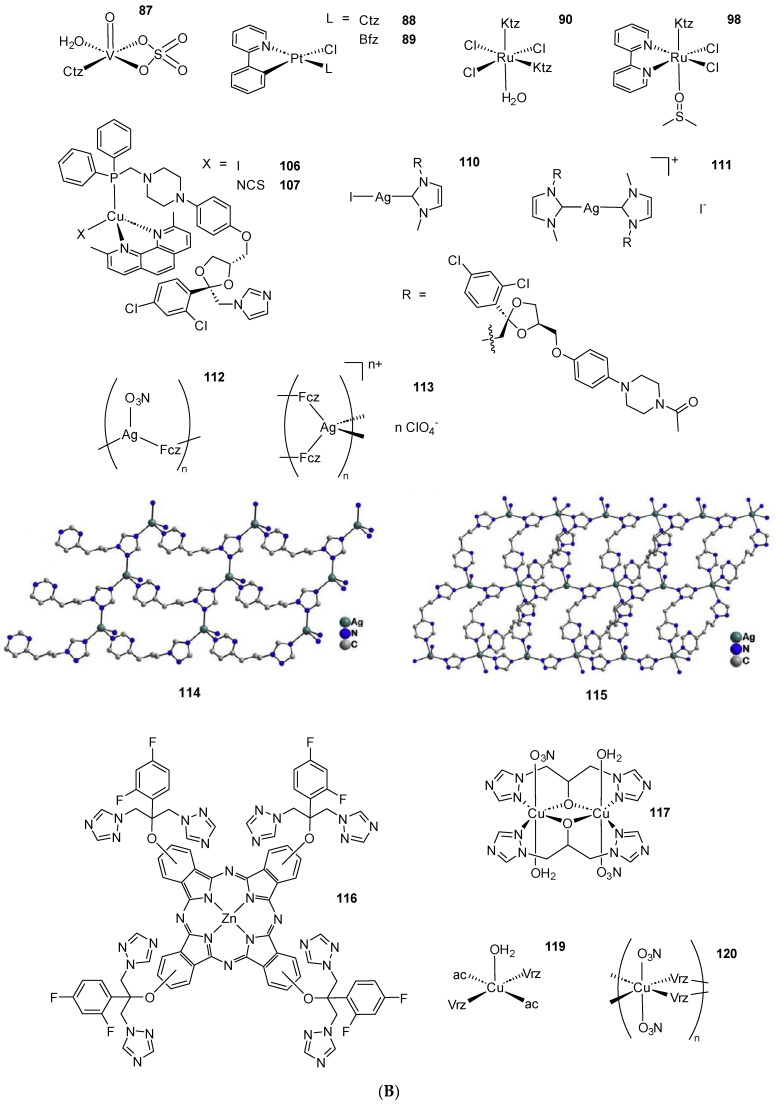

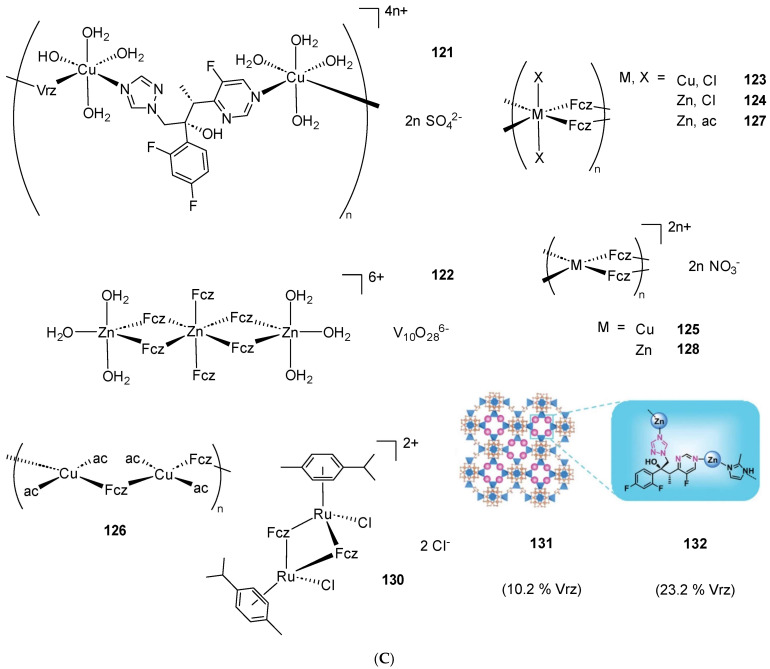
(**A**) List of selected complexes discussed in this work (unless otherwise drawn, the coordination with AA ligands always occurs via the *N*3 atom of the imz ring or the *N*4 atom of the triazole ring); (**B**) the schematic view of the 2D layered structure of compounds **114** and **115** were obtained with permission [50]; (**C**) the schematic representation of compounds **131** and **132** were obtained with permission [51].

The first study attempting to coordinate AAs from the Mcz family to a metal center was reported by Davis et al. in 1998. The authors described the synthesis of two square planar Ir(I) complexes bearing an *N*-heterocyclic carbene (NHC) derivative of Mcz and Ecz (**1** and **2**, respectively, Scheme 2). Crystals of **2** suitable for X-ray measurements were successfully grown, which helped deducing the structure of the Mcz analog, along with NMR spectroscopy measurements. The reasoning behind this work was the production of biologically relevant organometallic NHCs known to be involved in the metabolism of vitamin B1. However, no further biological investigations were carried out [49].

More than one decade later, Abd El-Halim et al. published seven new aqua chloro complexes of Mcz with a Co(II), Cr(III), Cu(II), Fe(III), Mn(II), Ni(II) or Zn(II) metallic core and tested them in vitro against several fungi and bacteria strains [52]. The authors supported the proposed structures by elemental and thermal analysis, IR spectroscopy, magnetic moment and molar conductance determination. However, these data constitute hardly convincing evidence, due to their indirect nature and the confusing deduction toward the formation of so-called chelates. Four species of fungi (*A. fumigatus*, *C. albicans*, *P. italicum*, *S. racemosum*) and four species of bacteria (*B. subtilis*, *E. coli*, *P. aeruginosa*, *S. aureus*) were selected to perform the biological experiments. Globally, most complexes showed similar or slightly better growth inhibition than the free AA. Exceptions are *C. albicans* and *P. aeruginosa*, against which almost no compound displayed the same activity as Mcz. However, the best improvements in growth inhibition were provided by Fe(III) and Ni(II) complexes against *B. subtilis* and by the Zn(II) complex against *E. coli* [52]. Following the same protocol, the authors published a similar study concerning Tcz. In this case, the increase in activity through complexation of the drug was more striking. Notably, promising results were given by Cr(III), Fe(III) and Mn(II) complexes against *A. fumigatus*, by all seven complexes against *C. albicans*, by Cr(III) and Mn(II) complexes against *P. italicum*, by Cr(III) and Cu(II) complexes against *S. aureus* and by all complexes against *B. subtilis* [53].

This work was criticized by Barba-Behrens and coworkers for an abusive usage of the word ‘chelate’ and a lack of spectroscopic data in support of the proposed molecular geometries. By contrast, the group of Barba-Behrens synthesized a similar library of Tcz complexes possessing a Cd(II), Co(II), Cu(II) or Zn(II) metal center and provided strong evidence for the proposed structures by means of NMR, IR and UV-Vis-NIR spectroscopy, as well as elemental analysis, molar conductivity and magnetic susceptibility measurements. Furthermore, the crystal structures of **3**–**6** (Scheme 2) were elucidated. Following characterization, the compounds were tested against three cancer cell lines (HCT15, MCF7 and HeLa). No or low activity of the complexes was observed against the two last cell lines, apart from a mediocre IC_50_ value of 13.542 µg/mL for **6** on HeLa. Once converted into micromolar and weighed according to the number of AA ligands in the coordination compound (wAA), this value becomes 27.067 µM (Table 1). Against HCT15 cells, four complexes showed similar activity compared to cisplatin, but only **3**, displaying an IC_50_ value of 3.10 µg/mL (6.78 µM wAA), was more active than this reference drug. In comparison, Tcz was not active against any of the three cell lines [54]. The same group expanded their series of Tcz derivatives by reporting the synthesis and cytotoxicity of eight new Ni(II), Pd(II) and Pt(II) complexes featuring this AA. These compounds were confirmed by NMR, IR, UV-Vis-NIR spectroscopy, ESI-MS, elemental and molar conductivity analysis. Additionally, crystals suitable for X-ray diffraction of **7** and **8** (Scheme 2) were successfully grown. The species were tested against the HCT15, HeLa, MCF7 and PC3 cell lines. Considering that Tcz did not show any measurable activity, the results revealed encouraging IC_50_ values below 100 µM. However, only **9** (Scheme 2) displayed better activity (15.14 µM wAA) than cisplatin (18.43 µM) against HeLa cells (Table 1) [55].

**Figure 3:**
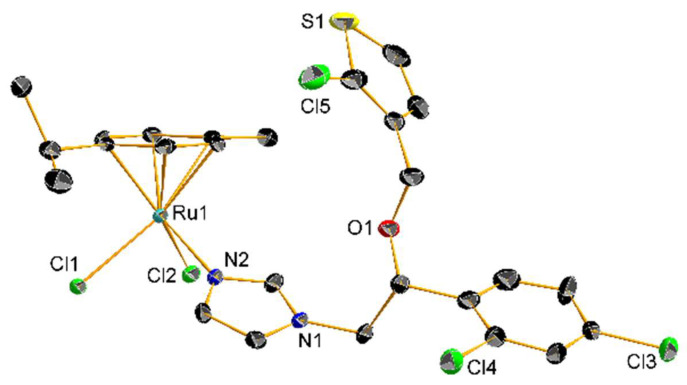
Crystal structure of **13** [72] with thermal ellipsoids drawn at the 30% probability level and hydrogen atoms omitted for clarity.

In 2013, Gasser and coworkers showed the importance of careful interpretation of the biological data of piano stool Ru(II) complexes possessing a coordinated monodentate drug. A considerable number of these compounds displayed very similar IC_50_ values against HeLa and L6 cell lines compared to an equimolar mixture of the free drug and the corresponding complex bearing a DMSO molecule instead of the medically relevant ligand. Further NMR analysis concluded that dissociation of the drug was occurring in DMSO-d6, questioning the relevance of data obtained for certain coordination compounds which are often stored in DMSO for biological purposes. Among all three AA complexes considered in this study, an Mcz derivative (**10**, Scheme 2) showed such decomposition, but still revealed better activity than the free AA. Moreover, the authors tested this compound against *T. Cruzi*. The slight differences obtained between the IC_50_ value of **10** and an equimolar mixture of the corresponding DMSO complex with the uncoordinated AA emphasized the partial nature of the dissociation [71]. Turel and coworkers synthesized the same Mcz complex **10**, along with the corresponding compounds in which one or two chlorides were substituted by the same AA ligand (**11** and **12**, respectively, Scheme 2), as well as all the Tcz (**13**–**15**, Scheme 2) and Ctz analogs. The resulting nine complexes were confirmed by NMR, UV-Vis, IR and HRMS analysis and, in particular, X-ray crystallography of **13**, which, at the time, was the first reported structure of a Tcz coordination compound (Figure 3). All complexes were tested against *C. lunata* and showed lower growth rate inhibition than the free AA. Interestingly, the antifungal potency of the compounds inversely correlated with the number of AA ligands. In addition, **10**–**12** were tested against the worm *S. mansoni*, but lethal toxicity toward this parasite appeared only at concentrations of 100 µg/mL [72].

Simpson et al. reported five new [2+1] *fac*-Mn(I) tricarbonyl complexes bearing an AA ligand and investigated their antimicrobial activity. The resulting species featured three carbonyl moieties, a monodentate AA ligand and a 2,2′-bipyridine (bpy) bidentate ligand derivative. These structures were confirmed by NMR, IR spectroscopy, as well as ESI-MS and DFT calculations. The compounds, among which two Mcz derivatives (**16** and **17**, Scheme 2), were tested against a series of four G+ bacteria (*S. aureus*, *S. epidermidis*, *E. faekalis*, *E. faecium*), four G− bacteria (*E. coli*, *P. aeruginosa*, *Y. pseudotuberculosa*, *Y. pestis*) and two kinetoplastids (*L. major*, *T. brucei*). Compound **17** yielded MIC values identical to or reduced by half compared to Mcz against G+ bacteria, while **16** gave more encouraging results, with MICs of 2.5 µM or 1.25 µM, which means between 2 times and 8 times lower than the ones displayed by the free AA (Table 2). Remarkably, the same compound was the only one of the series to show weak activity against G- bacteria, with the lowest measured MIC at 10 µM against *Y. pestis*. Moreover, this complex exhibited the lowest IC_50_ values against *L. major* and *T. brucei* as well (1.8 µM and 0.4 µM, respectively, Table 3). Unfortunately, comparison with its toxicity toward human cells gave mediocre selectivity indexes (Sis) [73].

The same year, Karaoun and Renfrew investigated the cytotoxic and luminescent properties of two Ru(II) Ecz complexes (**18** and **19**, Scheme 2). The compounds were characterized by NMR, UV-Vis and fluorescence spectroscopy, as well as mass spectrometry and elemental analysis. Interestingly, UV-Vis and ESI-MS analysis revealed that in the dark, an aqueous solution of **18** undergoes complete substitution of the chloride ligand by water within 24 h, inducing a blue shift in the absorbance spectrum. Conversely, **19** only showed the analogous behavior upon irradiation with green light, which lead to substitution of one Mcz, producing phosphorescence, as well as the same aqua complex and the free AA. The luminescent phenomenon and the photochemical decomposition were proposed to occur competitively both from the ^3^MLCT excited state, resulting in a turn-off phosphorescence response. With such properties, **19** was recognized as a potential photosensitizer for photodynamic therapy, and was thus tested on four cancer cell lines (MCF7, LNCaP, PC3 and DLD1), along with **18** and Ecz nitrate. The cytotoxicity of **19** remained relatively high in the dark, even exceeding that of the free AA occasionally (Table 1). However, **18** and **19** were found to be almost equally active when irradiated, with IC_50_ values ranging from 0.4 µM to 2.85 µM (5.70 µM wAA). The authors concluded that the potency of these molecules probably originates from the production of their aqua derivative, although the latter globally displayed lower efficiency, possibly because of poor cellular uptake [56].

No other coordination compound from this family of AAs was then reported until 2020, when Aziz et al. investigated the ability of three Cr(III) complexes, one of which designed with a Mcz ligand, to bind the insulin receptor for antidiabetic purposes. This work was based on combination of theoretical data furnished by the authors through spectra simulations, DFT calculations and molecular docking studies with experimental data in the form of IR, UV-Vis spectroscopy, mass spectrometry, TGA, molar conductivity measurements and DNA binding affinity tests. The obtained correlations, varying from moderate to excellent, provided evidence for the proposed structures. The potential of the complexes to bind the insulin receptor was assessed by calf thymus DNA (ctDNA) titration with each compound. The resulting hyperchromism observed by UV-Vis spectroscopy yielded a binding constant (K_b_) of 10^6^ M^−1^ for the Mcz derivative. Meanwhile, molecular docking calculations revealed the high affinity of this compound for the insulin receptor [88]. Adopting a similar dual theoretical–experimental approach, Hussien and coworkers reported the synthesis of three V(IV) inorganic compounds, among which **20** (Scheme 2), as well as their DNA binding ability and their cytotoxicity in vitro. Titration of ctDNA with **20** resulted in spectroscopic hypochromism. From this experiment, the authors deduced the affinity of the complex with DNA, supported by DFT calculations, and hypothesized an intercalation mechanism. However, the anticancer tests carried out against HepG2 and MCF7 cell lines showed IC_50_ values of 5.27 µM (10.5 µM wAA) and 2.98 µM (5.96 µM wAA), respectively, which is lower than cisplatin in both cases (25.5 µM and 19.0 µM, respectively). No comparison with the activity of the free AA was reported [57].

**Figure 4:**
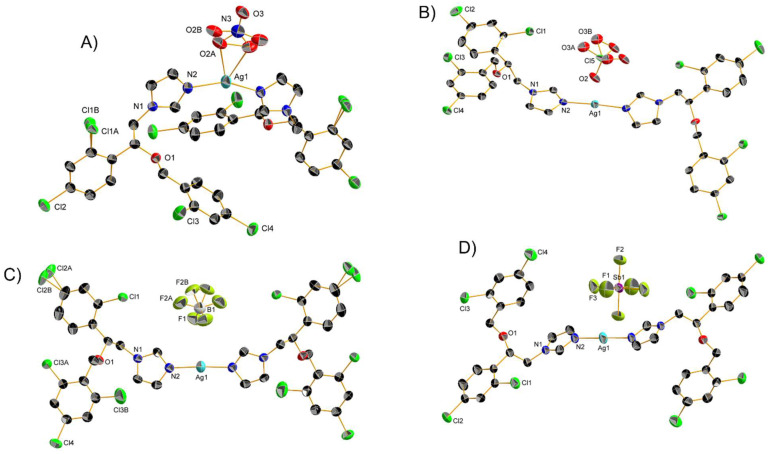
Crystal structures of (**A**) **21 [58]**, (**B**) **22 [58]**, (**C**) **23 [59]** and (**D**) **24 [59]** with thermal ellipsoids drawn at the 50% probability level and hydrogen atoms omitted for clarity.

Two Ag(I) Mcz complexes, namely **21** and **22** (Scheme 2), were studied by Ochocky and coworkers for their cytotoxicity. Crystals suitable for X-ray spectroscopy were obtained, and the resulting structures revealed distorted linear geometries (Figure 4A,B). Despite their similar molecular formula, these compounds exhibited notable structural differences, among which a N-Ag-N angle significantly lower than 180°, coplanarity of the two imz moieties and stronger interaction with the counterion in the case of **21**. The results suggested that **21** and **22** lie at extreme values within their group of analogs, considering the Ag-N and Ag-O bond lengths, as well as the N-Ag-N angle. In order to assess their anticancer properties, **21** and **22** were tested against HepG2 and non-tumoral Balb/c3T3 cell lines using four biochemical endpoints in order to determine their IC_20_ and IC_50_ values. The latter lay in the submicromolar range, while the free AA, as well as the corresponding silver salts, displayed no activity within the tested concentrations (Table 1). Unfortunately, moderate selectivity was only achieved at IC_20_, i.e., at a concentration where cisplatin is much more potent [58].

The author complemented their study with two new similar coordination compounds bearing a BF_4_^−^ and a SbF_6_^−^ counterion (**23** and **24**, respectively, Scheme 2). As determined by X-ray crystallography (Figure 4C,D), the latter displayed linear geometry like their ClO_4_^−^ analog **22** with an N-Ag-N angle of exactly 180° measured in the case of **24**. Moreover, **24** is the only compound of the series to possess an inversion center at the silver atom thanks to the peculiar relative position of the two Mcz ligands. Aware of the AA medicinal versatility, the authors decided to test the whole series of four complexes in vitro against G+ (*S. aureus*, *S.epidemnidis*, *M. luteus*, *B. subtilis*, *B. cereus*, *E. faecalis*) and G− bacteria (*S. typhimurium*, *E. coli*, *P. mirabilis*, *K. pneumoniae*, *P.aeruginosa*), as well as yeasts (*C. glabrata*, *C. albicans*, *C. parapsilosis*). For all compounds, better activity toward G+ bacteria than the corresponding Ag(I) salts was measured, but no significant increase compared to the potency of the free AA was observed, apart from the notable exception of **21**, with an MIC of 0.49 µM (0.98 µM wAA) against *M. luteus* (Table 2). Concerning G− bacteria, the opposite statement is true: that is, the free AA showed no activity and Ag(I) analog salts were the most active species. Meanwhile, the four complexes, particularly **21**, exhibited moderate activity against these pathogens. Finally, the most promising results were found in the case of *C. glabrata* and *C. albicans*, against which the four complexes showed MICs in the low micromolar range, lower than their corresponding Ag(I) salts or the free AA (Table 4) [59].

Stevanovic et al. published a recent study concerning AA coordination compounds of the Mcz family. In this work, an extensive biological investigation of seven square planar Au(III) trichloro complexes bearing an imz derivative ligand was carried out. Compounds **25** and **26** (Scheme 2) were characterized by NMR, UV-Vis and IR spectroscopy, as well as molar conductivity analysis. Additionally, crystals of **25** suitable for X-ray measurements were successfully grown. The authors first tested these compounds against different *Candida* strains (*C. albicans*, *C. parapsilosis*, *C. glabrata*, *C. krusei*, *C. auris*) and healthy MRC5 human cells. Globally, the two complexes yielded lower MIC values (between 0.4 µM and 18.3 µM) than their corresponding AA toward the pathogens (Table 4). Namely, **26** showed a more than 20-fold improvement in activity against *C. krusei* compared to Tcz. The same compound exhibited submicromal MIC values against *C. albicans* and *C. parapsilosis*, but was relatively toxic toward healthy cells (IC_50_ value of 6.5 µM). Conversely, **25** showed a higher IC_50_ value against MRC5 cell line (26.3 µM) than any measured MIC value toward the yeasts. Against G+ (*S. aureus*, *S. aureus* MRSA) and G− (*E.coli*, *P. aeruginosa*) bacteria, the potency gap between the two complexes and their corresponding AA was even more striking. However, the MIC values were generally higher than the ones measured against fungi, ranging from 9.1 µM to 219 µM (Table 2). The best activity was found against *P. aeruginosa* and especially *S. aureus* MRSA, which is surprising, given the resistant nature of this strain. Furthermore, **25** and **26** showed the capacity to inhibit *P. aeruginosa* pyocyanin production. These two complexes were even active at lower concentration and revealed significantly better results when diluted from 20 µg/mL to 10 µg/mL. This interesting feature was used in a last assay combining *P. aeruginosa* with A549 cancer cells in which the two compounds decreased cell death by approximately 15% at a 5 µg/mL (7.3 µM) concentration of **25** and a 2.5 µg/mL (3.6 µM) concentration of **26** [74].

### 2.2. Coordination Compounds of the Ctz Family of AAs

Shortly after the discovery of Mcz, Ctz was patented by Bayer in 1969 and approved for treatments in 1973 [97]. In 1974, the same brand developed Bfz, which was used for medical applications starting in 1983 [48]. Although considered members of the first AAs generation, Ctz and Bfz share little structural similarities with Mcz (Scheme 1). Indeed, they feature a more symmetrical structure, which makes them ligands of choice for exploring the inorganic and organometallic chemistry of such derivatives. In particular, the achirality of Ctz, a unique property among AAs [11], ensures a minimum amount of different stereoisomers throughout the synthesis and could thus explain its popularity over Mcz derivatives for coordination reactions.

In 1993, Sanchez-Delgado et al. reported the MDS phenomenon observed in the case of a Ru(II) Ctz complex against *T. Cruzi*. This pioneer work marked the beginning of a series of publications involving the coordination of Ctz, chloroquine and later Ktz onto various metal centers [78,79,98,99,100,101,102]. In their first paper, the authors proposed the peculiar 14-electron structure of **27** (Scheme 2), based on NMR analysis and computational investigations. Indeed, the corresponding 18-electron octahedral complex, bearing two additional solvent ligands, was calculated as less stable because of steric hindrance. Biological assays showed that the complex was approximately 10 times more potent against *T. Cruzi* epimastigotes than the free AA, with an EC_50_ of 0.1 µM (0.2 µM wAA, Table 3), and that a trypanocidal effect through cell lysis occurred after 96 h. When the amastigote form of *T. Cruzi* was grown on mammalian Vero cells, **27** exhibited less undesired toxicity than Ctz, and its EC_50_ decreased to 0.005 µM (0.01 µM wAA) [78].

This work was complemented in the fourth paper of the series by further biological investigations on **27** and comparison of its in vitro activity with five Au(III), Cu(I), Pt(II), Rh(I) and Ru(II) analogs. The complexes were characterized by NMR spectroscopy and, for **28** and **29** (Scheme 2), by X-ray crystallography. Compared to the results displayed by **27** against *T. Cruzi*, all other complexes performed worse, showing even less trypanostatic potency than the free AA in the case of certain compounds. These results were especially surprising regarding Ru complexes for which the cationic nature was designed to improve the activity of **27**, thanks to better solubility in biological medium and higher affinity with the cell membrane. In order to rationalize these data, the authors proposed that the potency of **27** relied on its labile chloride ligands. Subsequent studies involving this compound indicated that it exerts slightly inferior sterol inhibition compared to Ctz, which implies a distinct mechanism of action. After comparative HPLC experiments in the presence of DNA, the authors concluded that the inorganic compound probably decomposed in the aqueous environment, leading to the dissociation of Ctz and the formation of a chlorinated metallic core able to bind DNA. This hypothesis could explain the superior antikinetoplastid capacity observed for **27** [79].

A new SAR study of the latter was published by the same group who introduced two new paramagnetic Ctz and Ktz Ru(III) complexes. The corresponding structures were confirmed by EPR and NMR spectroscopy by correlation with other data on similar compounds available in the literature. Compound **30** (Scheme 2) proved less active against *T. Cruzi* than its Ru(II) analog **27**. It was, in fact, the least potent complex of the series, although it displayed a slightly higher inhibition capacity than free Ctz. In regard to their previous work, the authors hypothesized that the Ru(III) core was less prone to hydrolysis, because of either a lower dissociation rate or the necessity of an initial reduction to Ru(II) in the medium, which results in a lower DNA binding affinity [100]. This series of publications on AAs was finally closed with the synthesis of six novel Ctz and Ktz complexes possessing a Cu(II) or Au(I) metal center. These species were characterized by NMR, UV-Vis and IR spectroscopy, as well as elemental analysis. Additionally, complex **31** (Scheme 2) was analyzed by X-ray diffraction and, along with the other paramagnetic compounds, by EPR spectroscopy and DC magnetic susceptibility determination. The biological tests on *T. Cruzi* revealed an almost identical activity for each Ctz coordination compound, ranging from 66 to 67% inhibition at 1 µM. By contrast, the free AA showed no inhibition at this concentration. With the exception of the well-known complex **27** (82.4% inhibition at 1 µM [100]), these surprisingly similar results suggest that the number of Ctz ligands, the metal atoms in the complex or even their oxidation states played no significant role in the observed activity. A possibility, in order to rationalize these data, consists of considering only the protecting effect of complexation that shields the drug from the biological environment, even though it seems unsound to discard the medical relevance of the metal core [101].

In addition to its antitrypanosomal activity, **27** and its close derivatives were later investigated in various biological assays. In particular, Rieber and coworkers reported the cytotoxic potential of this compound and its Ktz analog. With no synthetic or spectroscopic data provided, the authors tested the Ctz coordination compound **27** against C8161 melanoma monolayers. The IC_80_ value of the complex was measured at less than 50 µM (Table 1), and its capacity to initiate caspase-3-dependent apoptosis was investigated by means of immunoblotting assay. Although PARP fragmentation was observed, the results concerning the detection of activated caspase-3 in C8161 melanoma and HT29 carcinoma cell lines seemed negative. Therefore, further anticancer studies on **27** were abandoned in favor of the Ktz derivative, which will be discussed later in this review [103]. The same year, Navarro et al. tested some of their already published derivatives [79,100,101] and new AA coordination compounds against *S. cerevisiae*. The MIC values of the Ctz complexes remained similar to that of free Ctz (1 µM). Further experiments assessing the toxicity and influence of these species toward human Neutrophils were performed. The phagocytes were first activated to display luminescence and their inhibition by the Ctz derivatives was assessed, yielding IC_50_ values between 4.4 µM and 19.7 µM. Because the latter were significantly higher than their corresponding MICs, and because the trypan blue exclusion method showed that none of the compounds was significantly cytotoxic toward Neutrophils below 100 µM, it was determined if a 0.5 µM concentration of the complexes could enhance the activity of the phagocytes. This was, however, not the case for the Ctz coordination compounds [104].

Turning their interest to the Pd(II) metal core, the authors later assessed the cytotoxicity of compound **32** (Scheme 2). The AA derivative was extensively characterized spectroscopically and displayed a square planar geometry by X-ray crystallography. Unfortunately, its IC_50_ values against PANC1, SKBR3, MDAMB231 and HT29 cancer cell lines were found to be 2 to 4 times higher than free Ctz [105]. This work was later completed by the investigation of two Pt(II) Ctz complexes for their cytostatic properties. Compounds **33** and **34** (Scheme 2) were confirmed by NMR, UV-Vis, IR spectroscopy, as well as elemental analysis and molar conductivity measurements. A *trans* configuration was proposed for both species, mainly based on the interpretation of IR data. By titration of the compounds with ctDNA, the authors observed a hypochromic response, which, together with increase in complex luminescence and of DNA melting point, implied DNA binding. Subsequent investigations through viscosity determination leaned toward affinity for the minor groove, and according to successful complex precipitation from the mixture, this interaction with DNA appeared as non-covalent.

Despite their binding ability, **33** and **34** displayed no toxicity in vitro against any of the six tested cell lines (PC3, MCF7, SKBR3, HT29, LoVo, B16/BL6). However, their ability to inhibit cancer cell growth was assessed and GI_50_ values between 5.5 µM and 25.8 µM (MCF7, SKBR3, HT29, B16/BL6) or higher (PC3, LoVo) were obtained. By contrast, Ctz and cisplatin induced cytotoxicity and systematically showed similar or significantly lower GI_50_ [106]. In an attempt to repurpose these compounds and already published ones [101], the same group tested eight Au(I), Cu(I) and Pt(II) complexes of Ctz and Ktz against fungi of the *Sporothrix* genus. The three new complexes of this series were characterized by NMR, IR, UV-VIS spectroscopy, ESI-MS and molar conductivity. All four Ctz complexes considered in the biological study exhibited MIC and MFC values against *Sporothrix schenckii* similar to the ones of the free AA, with the exception of **33**. As a consequence, the latter was discarded for subsequent measurements of MFC cumulative percentages against six fungal pathogen isolates, two *Sporothrix schenckii*, two *Sporothrix brasiliensis* and two *Sporothrix globosa* ones, in which the three metallic derivatives were able to kill 100% of the fungi population at concentrations below 40 nM. In particular, compound **35** (Scheme 2) displayed the best results, although it is worth noting that the gold salt AuClPPh_3_ yielded low MIC and MFC values as well, which indicated the possible biological relevance of the phosphine ligand (Table 4). However, the two Ctz complexes bearing such a ligand (**35** and **36**, Scheme 2) were proven by SEM to induce cell shape alteration in *Sporothrix schenckii*. Finally, the toxicity of these complexes against L929 and human red blood cells was assessed. No effect was observed on mammalian cells below the micromolar range, yielding higher to much higher SIs than uncoordinated Ctz, which was calculated at 2536 with respect to L929 cells and at more than 26,042 with respect to human red blood cells [89].

In parallel to the research involving medicinal analysis of **27** and its parental compounds, other contributions focused on piano stool Ru(II) complexes in order to overcome the limitation of this promising antiparasitic agent. For example, Sanchez-Delgado and coworkers attempted to synthesize potent analogs of this molecule with improved water solubility by assessing the antikinetoplastid properties of three octahedral and five piano stool coordination compounds bearing one or two Ctz ligands attached to a Ru(II) or Ru(III) metal center. The structure of each compound was determined by NMR and IR spectroscopy, as well as elemental analysis. Additionally, all piano stool species (**37–40**, Scheme 2) were confirmed by X-ray crystallography. Globally, the complexes showed lower LD_50_ values against the two tested parasites (*L. major*, *T. cruzi*) than the free AA. However, **40** yielded particularly promising results, as well as low toxicity toward mammalian cells (Table 3).

**Figure 5:**
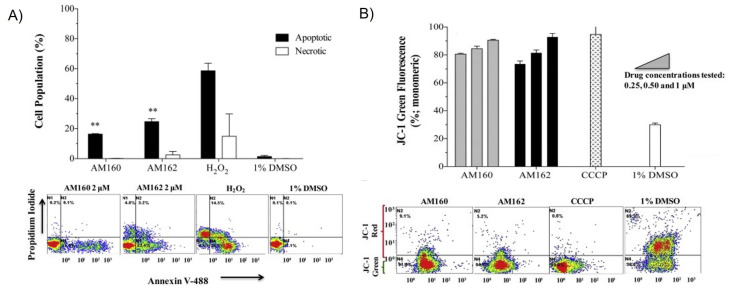
(**A**) Percentage of apoptotic and necrotic parasites treated with **37**, **40** and controls (**upper**) and corresponding flow cytometry scatterplots (**lower**). (**B**) Percentage of JC-1 green fluorescent parasites treated with **37**, **40** and controls (**upper**) and corresponding flow cytometry scatterplots (**lower**); obtained and modified with permission [107].

Another compound, namely **37**, displayed particularly striking results with LD_50_ values of 0.015 µM against *L. major* promastigotes, 0.1 µM against *T. cruzi* epimastigotes and an IC_50_ value over the selected range toward human osteoblasts. Altogether, these data resulted in an SI calculated at more than 500 and more than 75 in the case of *L. major* and *T. cruzi*, respectively. Further analysis of *L. major* amastigotes grown in mice macrophages, revealed an IC_70_ value of 0.029 µM, acknowledging **37** as a promising antiparasitic agent [80]. Four years after this work, in vivo experiments against *L. major* with the two most promising complexes of the series, namely **37** and **40**, were performed. The inhibition of lesion size growth in BALB/c mice revealed that the latter was significantly more efficient than the free AA at fighting the infection. Furthermore, the same compound considerably decreased the rate of lesion expansion in infections, with a 10 times higher concentration of pathogens, and was proven to decrease parasite load by at least 50% by means of PCR analysis of mouse DNA extract. During the whole in vivo study, no sign of toxicity ascribable to the complexes was observed. In order to investigate the mechanism of action, the authors carried out flow cytometry measurements on *L. major* promastigotes treated with **37** and **40** using a dual annexin V-propidium iodide probe (Figure 5A). This method enabled discrimination between apoptotic, necrotic and healthy cells through the detection of external phospholipids independently of membrane rupture. The results revealed almost negligible necrosis and a high percentage of apoptosis, whose origin was identified as the depolarization of mitochondrial transmembrane by subsequent flow cytometry analysis using the JC-1 reagent. Indeed, the fluorescence shift from red to green indicated the inability of the probe to aggregate in the mitochondria (Figure 5B). The presence of a later apoptosis biomarker, namely endonuclease-mediated DNA digestion, was controlled by terminal deoxynucleotidyl transferase dUTP nick end labeling assay and by assessment of the cell population carrying fragmented DNA. Both experiments confirmed the apoptotic pathway [107].

Deviating from the traditional *p*-cymene (cym) ligand, Rodriguez Arce et al. provided another contribution in the field of piano stool Ru(II) complexes with the synthesis as well as in vitro antikinetoplastid and antitumoral properties assessment of **41** (Scheme 2). This compound was characterized by NMR, IR spectroscopy, molar conductivity and elemental analysis. Activity measurements against *T. cruzi* and *T. brucei* yielded IC_50_ values in the nanomolar range (0.25 µM and 0.6 µM, respectively), much lower than the ones of the free AA, while toxicity against healthy J774 cells arose with an IC_50_ at 1.9 µM (Table 3). The similarly excellent activity toward the two parasites, despite their completely different dependence on ergosterol, led to the hypothesis of a distinct mechanism of action of **41**. Accordingly, further analysis was carried out in order to determine if this complex proceeds, like Ctz, by lanosterol C_14_-demethylase inhibition. Comparative TLC of *T. cruzi* lipid extracts upon treatment with the complex confirmed the accumulation of squalene instead, which is an earlier precursor in the biosynthesis of ergosterol and cholesterol. Compound **41** was additionally tested against the cancerous A2780, MCF7 and HeLa cell lines, giving lower IC_50_ values (0.54 µM, 5.4 µM and 3.6 µM, respectively) than the free AA or even cisplatin (Table 1). Interestingly, the titration of a ctDNA-ethidium bromide (EB) mixture with this organometallic compound showed no quenching of fluorescence, implying an at best weak affinity with DNA and thus excluding DNA interaction as main mode of action to explain its cytotoxicity and antitrypanosomal activity [61].

Navarro and coworkers investigated the same kind of triphenylphosphine-containing species for their antiparasitic activity. For this purpose, four organometallic piano stool Ru(II) AA derivatives—among them the Ctz derivative **42** (Scheme 2)—were synthesized and tested against *L. amazonensis*. Their structure was confirmed by NMR, FT-IR, UV-VIS spectroscopy, ESI-MS and molar conductivity analysis. After 48 h incubation, **42** yielded IC_50_ values of 0.57 µM and 0.02580 µM against *L. amazonensis* promastigotes and amastigotes, respectively (Table 3). By comparison, the IC_50_ value of uncoordinated Ctz lay at 0.87 µM against the promastigote form. The parasite amastigotes were cultivated in murine macrophages whose viability was shown to be affected only in the micromolar range. Moreover, the infection ratio of the macrophages was found to already significantly decrease upon treatment at less than half of the IC_50_. Concerning ultrastructural alterations on promastigote cells, SEM and TEM could reveal the appearance of membrane protrusions, shortening of the flagellum, loss of the mitochondrial matrix, disorganization of kDNA structure and anomalous chromatin condensation after exposition to **42** at 0.3 µM or 0.5 µM [81].

The interest in piano stool Ru(II) complexes was not restricted to antikinetoplastid research. Indeed, the group of Sanchez-Delgado investigated six already published [80] Ctz derivatives along with six Ktz ones for their cytotoxic properties. The first biological experiments were performed on five cancerous (DU145, 22Rv1, LNCaP, YT, HeLa) and three healthy (HaCaT, MCF10A, MEFs) cell lines. While complexation of the AA globally afforded an increase in activity concerning **37**, **43** and **44** (Scheme 2) against LNCaP, YT and HeLa cells, all the Ctz coordination compounds displayed higher CC_50_ values than the free AA against DU145 and 22Rv1, with the single exception of **43** against 22Rv1 (Table 1). Moreover, the same complex yielded the most promising result of the whole series in the form of a CC_50_ at 2.4 µM (4.8 µM wAA) against LNCaP, which corresponds to an SI of more than 128. Generally, the latter lay above 1 for most of the Ctz complexes, because the high CC_50_ values obtained against HaCaT cells were taken as reference. However, much higher toxicity was unfortunately measured toward MEFs cells. The authors subsequently analyzed YT cells exposed to the species by means of flow cytometry. The resulting data enabled a quantitative discrimination between apoptosis and necrosis to be made and thus emphasized the probable difference in the mechanism of action exhibited by Ctz compared to that of its coordination compounds. Namely, the tumoral cells clearly elicited an apoptotic pathway with Ctz, while necrosis was found to be more dominant upon treatment with certain complexes, especially **37** and **38**. Finally, the binding affinity for DNA was assessed by means of CD spectroscopy and thermal denaturation analysis of ctDNA.

Compound **45** (Scheme 2) was the only Ctz derivative found to significantly impact the CD spectrum, which implies a strong interaction with DNA either by covalent groove binding or by intercalation. The former was concluded by observation of a slight decrease (2.3 °C) in the melting temperature of ctDNA after mixing it with **45**, and the amount of bound Ru was calculated at 585 nmol/mg DNA by the Burton assay [60]. Turel and coworkers reported the antifungal properties of **37**, along with two other piano stool Ru(II) complexes bearing two and three Ctz ligands instead of chlorides substituents (**46** and **47**, respectively, Scheme 2). After successful characterization of **46** by X-ray crystallography, biological assays against *C. lunata* displayed the same tendency observed for the Mcz and Tcz analogs **10–15**, that is, a growth inhibition potency inversely correlated with the number of Ctz ligands and significantly lower than that of the free AA [72].

**Figure 6:**
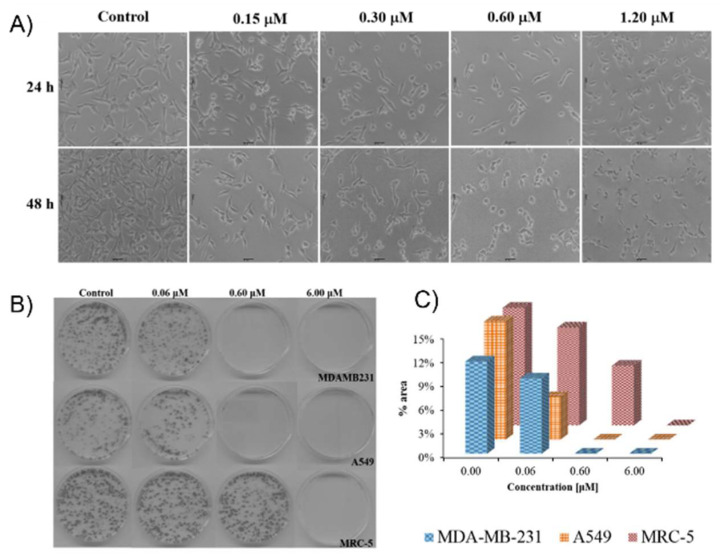
(**A**) Microscopy images representing cellular morphology of MDAMB231 cells untreated and treated with different concentrations of **42**. (**B**) Colonies of MDAMB231, A549 and MRC5 cells untreated and treated with different concentrations of **42**. (**C**) Corresponding graphical representation of colonies area; obtained and modified with permission [62].

With other collaborators, the group of Navarro published a DNA binding and anticancer study on their four piano stool Ru(II) coordination compounds bearing AA moieties [81]. In particular, the ability of complex **42** to quench blood human serum albumin (HSA) luminescence was assessed by fluorimetric titration. The results indicated a moderate affinity for the biomolecule with probably one single binding site. Subsequent titration with ctDNA resulted in a bathochromic shift and a decrease in UV-VIS absorbance. Intercalation was excluded as the binding mode, because no changes were detected during viscosity measurements of ctDNA upon the addition of the coordination compound. The inability of **42** to alter DNA helicity was further confirmed by CD spectroscopy. However, gel electrophoresis assays revealed its capacity to displace EB, which is uncommon for non-intercalative molecules. These confounding conclusions were solved by performing a competitive affinity analysis between the organometallic compound and Hoechst 33258, a known minor groove binding dye. Titration of **42** into a mixture of ctDNA and Hoechst 33258 yielded a significant decrease in fluorescence intensity, which confirmed the minor groove interaction mode. Nevertheless, previously discussed results suggested weak binding ability, which was further proved by NMR spectroscopy tests with guanosine. Compound **42** was tested against cancerous A549, DU145, MDAMB231 and non-cancerous MRC5, L929 cell lines. The resulting IC_50_ values, in the nanomolar or lower micromolar range, indicated better cytotoxic capacity than the free Ctz (Table 1). Therefore, the coordination compound was incubated with MDAMB231 cells in order to study its effect on cancer cell morphology. Most of the originally spindle-shaped cells became spherical at a concentration around the IC_50_ value (0.63 µM, Figure 6A). Moreover, the complex showed its ability to prevent A549 and MDAMB231 cells colony formation at 0.6 µM, whereas it only displayed similar activity at a 10-fold higher concentration for healthy MRC5 cells (Figure 6B,C). Subsequent analysis employing the Boyden chamber assay revealed that **42** was able to almost completely inhibit MDAMB231 cells migration at a concentration of 0.6 µM. Finally, the author afforded evidence toward a concentration dependent apoptotic pathway by means of cell cycle and cytometry analysis with Annexin V [62].

From the beginning of the present section, seven publications concerning the field of piano stool Ru(II) complexes have been discussed. However, the already discussed communication of Gasser and coworkers cautioning the scientific community against the lability of *N*-heterocyclic ligands in such coordination compounds featured the investigation of compound **37**. Similarly to its Mcz analog **10**, the latter displayed partial dissociation in DMSO, which was followed by NMR spectroscopy and illustrated by comparison of its biological activity with that of the free AA mixed with [Ru(DMSO)(cym)Cl_2_]. Indeed, the obtained IC_50_ values were close: 3.1 µM and 3.5 µM against Hela cell line, 1.6 µM and 2.1 µM against L6 cell line, 0.008 µM and 0.017 µM against *T. cruzi*. The similarity of these biological data again demonstrated the questionable nature of certain conclusions found in the literature, especially when no stability measurements are provided by the authors [71].

Before their above mentioned works on Mcz [52] and Tcz [53], Abd El-Halim et al. synthesized Co(II), Cr(III), Cu(II), Fe(III), Mn(II), Ni(II) and Zn(II) complexes of Ctz and tested them against various microbial organisms. The general procedure regarding characterizations and biological analysis of the molecules was identical to the one adopted in the authors’ subsequent studies. Globally, the antibacterial results displayed by the compounds were mixed. Even though the complexes showed globally similar or inferior activity compared to the free AA, significantly higher inhibition potency against *P. italicum* was observed for the Cr(III), Fe(III), Mn(II) and Zn(II) coordination compounds. Moreover, the latter species displayed significant activity against *B. subtilis* and *E. coli* as well [108]. Barba-Behrens and coworkers produced a vast library of sixteen similar Ctz coordination compounds based on a Co(II), Cu(II), Ni(II) or Zn(II) core and assessed their anticancer properties. These complexes were designed mainly with chloride, bromide and nitrate ligands, resulting in various geometries depending on the metal center. The latter were determined by IR, UV-Vis, UV-Vis-NIR reflectance spectroscopy, elemental analysis, magnetic susceptibility and molar conductivity measurements. Additionally, Zn(II) complexes were studied by NMR spectroscopy and crystals of **48**–**52** (Scheme 2) suitable for X-ray diffraction were successfully grown. The authors decided to first follow the ability of the three Zn(II) complexes to bind guanosine monophosphate by ^1^H NMR spectroscopy and pH measurements. The chemical shifts and pH changes observed in aqueous solution confirmed significant affinity for the nucleotide and thus potential DNA binding capacity. However, this interaction took place relatively slowly, reaching equilibrium after more than one day, which was probably a consequence of the requirement of initial hydrolysis. The cytotoxic potency of the complexes was tested against three different cell lines (HeLa, PC3, HCT15). Even though the compounds globally showed better or slightly worst activity than the free AA, only two of them (**53** and **54**, Scheme 2) yielded efficiency comparable to cisplatin against Hela (Table 1).

**Figure 7:**
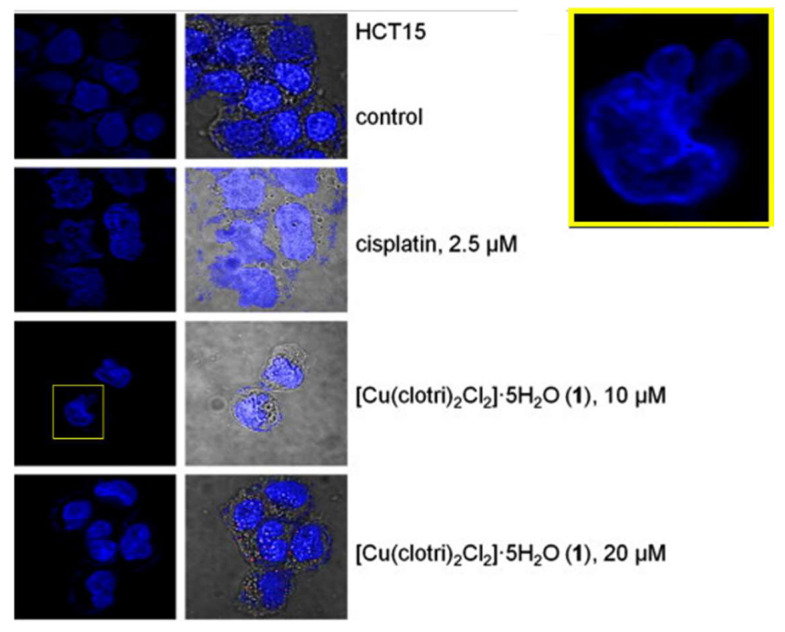
Confocal microscopy images of HCT15 cells untreated, treated with cisplatin and treated with different concentrations of **48** (**left**) and amplification emphasizing chromatin condensation and nuclear membrane fragmentation (**right**); obtained and modified with permission [63].

Interestingly, the lowest IC_50_ values were displayed by three of the Cu(II) complexes (**48**, **53** and **54**), and that is why subsequent analyses were carried out on compounds **48** and **54**. The former was shown by confocal microscopy to induce chromatin condensation and even early nucleus fractionation in HCT15 cells (Figure 7). Furthermore, treatment of HeLa cells with **48** and **54** caused a significant disturbance of apoptosis markers (Ki-67 protein, Bax, Bcl-2, caspase-3, caspase-9 antibodies) expression. Namely, Ki-67 expression quickly dropped to 0 with increase in complex concentration, as expected for this cell proliferation biomarker. Bax and Bcl-2 expressions exhibited the same behavior, which was explained by the quick defensive response of the tumoral cell, resulting in the early presence of Bcl-2 competitive with the Bax apoptosis marker. Finally, the increasing expression of caspase-3 and -9 suggested cell apoptosis, even at lower concentrations. Additional evidences toward cell death upon treatment with these two Cu(II) complexes were supplied by terminal deoxynucleotidyl transferase dUTP nick end labeling assay [63].

**Figure 8:**
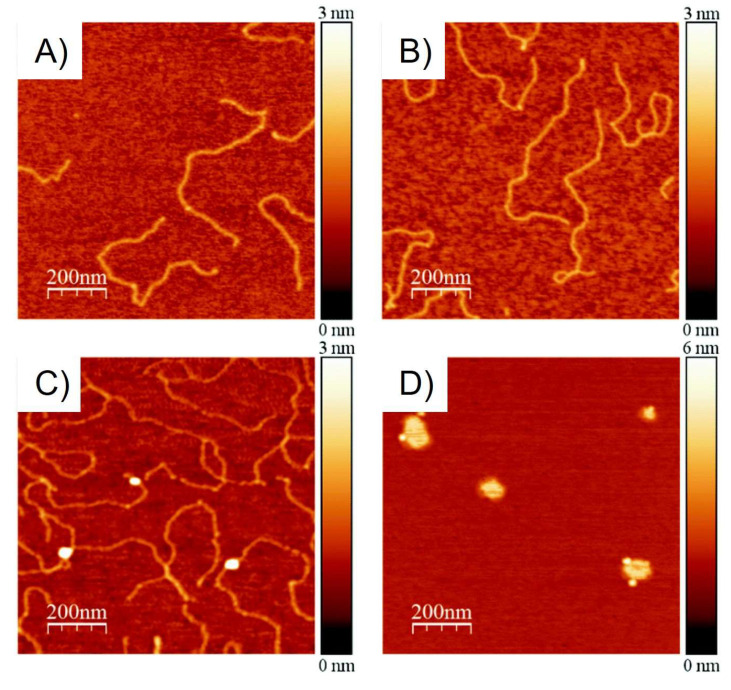
TM-AMF images of linearized pUC19 plasmid DNA mixed with **48** in different ratios DNA:complex (**A**) 1:0, (**B**) 10:1, (**C**) 10:3 and (**D**) 2:1; obtained and modified with permission [109].

From this work, eleven complexes were selected in order to elucidate their mode of action through various analysis. Namely, the log*P* values were measured in order to determine the lipophilicity of these species. It was found that none of the tested compounds exhibited a non-negative log*P* value, even though no correlation with the previously published cytotoxicity data could be concluded. According to a minor decrease in the melting temperature of ctDNA (0.5 °C) and fluorescence quenching of a ctDNA-EB mixture, certain coordination compounds were regarded as potential weak DNA binders. Subsequent titration of ctDNA with these complexes followed by LD spectroscopy showed the appearance of new signals in the region around 260 nm. As no major disturbance of linearity was observed, intercalation or groove binding was supposed. However, the former was discarded due to the presence of weak positive signals, which are not expected in in case of intercalation. The DNA binding mode was further determined to induce DNA bending, but not kinking, because only minor changes were obtained during titration monitored by CD spectroscopy. These dichroism results were confirmed by TM-AFM as well, illustrating the resulting linearized plasmid DNA coiling upon treatment with complex **48** (Figure 8). Additional proof was afforded by ICP-MS measurement for metal content of isolated HeLa cell DNA adducts. Indeed, the observed tendency was found to be very similar and in particular, the three Cu(II) complexes (**48**, **53** and **54**) displayed the highest DNA affinity.

Moreover, the same complexes yielded electrochemical potentials lying between −0.082 V and 0.117 V for the Cu^2+/+^ reduction, in a range within which hydroxyl radical generation probably occurs in biological systems thanks to H_2_O_2_ availability. Comparatively, all other complexes tested by CV did not show any potential with absolute values lower than 0.1 V. The hypothetic ability of the Cu(II) coordination compounds to exert oxidative DNA damage was proven by gel electrophoresis, as an increasing concentration of complex **54** caused a more pronounced fading of plasmid DNA with a corresponding IC_50_ value calculated at 10.47 µM. Despite the seemingly arbitrary selection of compounds for each type of analysis, the authors could finally rationalize the disparity obtained in the cytotoxicity data of their previous study by emphasizing the superior capacity of Cu(II) complexes to produce reactive oxygen species (ROS) in the vicinity of DNA [109].

Among the five Mn(I) tricarbonyl complexes of AAs reported in the above-mentioned work of Simpson et al. [73], the Ctz derivative **55** (Scheme 2) is the only one characterized by X-ray crystallography. This compound exhibited the most promising antibacterial activity of the library with MIC values of 0.625 µM against *Staphylococcus* species, even though it was found to be almost inactive against G− bacteria (Table 2). The biological tests against kinetoplastids yielded results similar to the Mcz analogs **16** and **17**, with IC_50_ values in the low micromolar or nanomolar range, but poor SIs (Table 3) [73]. The same molecule with a different conterion (**56**, Scheme 2) was selected as the main scaffold by Mendes et al. [75] in their SAR study on CO-releasing molecules. The authors reported the synthesis and antibacterial activity of six Mn(I) and one Re(I) coordination compounds of Ctz, which were obtained in two steps from the metallic bromopentacarbonyl species. The structures were determined by NMR, FT-IR spectroscopy, HR-MS and elemental analysis. The molecules were tested against one G+ (*S. aureus*) and two G− (*E. coli*, *S. enterica*) bacteria. In the case of *S. aureus*, the resulting MIC values lay 4.5 to 23 times lower than that of free Ctz for complexes **56**–**59** (Scheme 2, Table 2). The four latter even showed slight antiproliferative properties against G- bacteria. The best compound, the Re(I) species **57**, was further tested against ten strains of G+ and G- bacteria along with [Re(CO)_3_(bpy)Br] and the uncoordinated AA in order to highlight the synergistic feature of drug complexation.

The three remaining organometallic molecules displayed less promising results, especially **60** (Scheme 2), which was even unable to inhibit the growth of *S. aureus* colonies. The authors pointed out the probable role of steric hindrance at the 4 and 4′ positions of the diimine moiety in order to explain such a loss of bioactivity, whereas functionalization at different positions and replacement of bpy-like ligands for imz derivatives, or even NHCs, resulted in a less negative impact. Apart from this innocuous compound, the six other complexes were incubated with *S. aureus* and their CO-releasing ability was confirmed by COP-1 probe [110] experiments by fluorescence microscopy. Furthermore, the common problem of CO-releasing molecules decomposition, promoted in blood environment by hemoglobin scavenging, was discarded by blood oximetry measurements.

**Figure 9:**
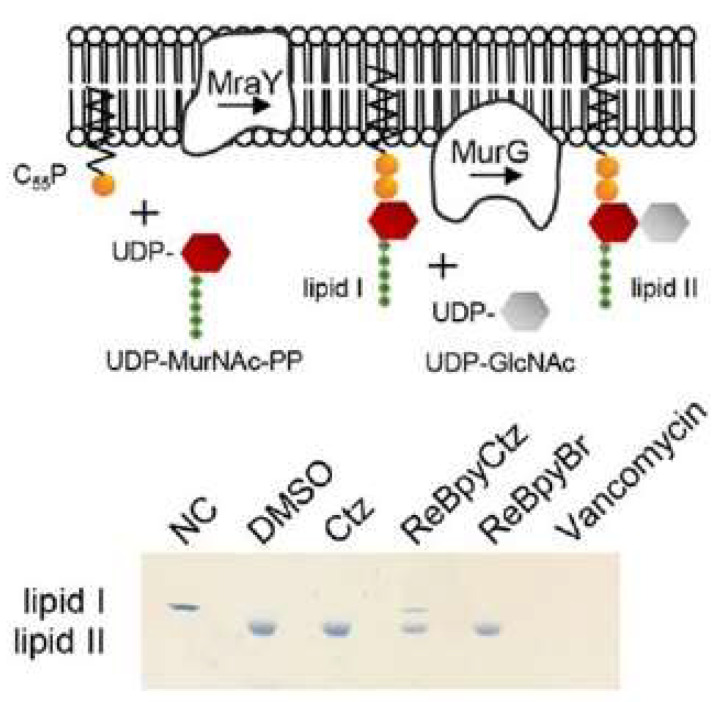
Schematic representation of lipid II synthesis in *S. aureus* (**upper**) and TLC plate of a medium containing lipid I, purified recombinants for the MurG-mediated reaction and different compounds (**lower**); obtained and modified with permission [75].

Additional understanding of the mechanism of action was provided by extensive analysis of biological effects triggered upon treatment with **57**. Bioreporter profiling revealed the induction of cell membrane stress upon exposure of *B. subtilis* to this complex. Fluorescence microscopy employing various dyes could exclude membrane potential disturbance or alteration of its barrier function. This result led to the logical consideration of peptidoglycan biosynthesis inhibition and indeed, **57** was proven to cause peptidoglycan precursors accumulation in the cytoplasm. However, a fluorescence pulse labelling experiment revealed that uncoordinated Ctz was in fact significantly more potent at hindering early peptidoglycan synthetic activity, suggesting a distinct mechanism of action for the organometallic analog **57**. The use of purified components demonstrated the selective inhibition of the MurG-promoted formation of lipid II from lipid I (Figure 9). Moreover, **57** was shown to possess higher affinity for the peptidoglycan precursors than for the enzyme binding site, while Ctz displayed preference for the membrane carrier C_55_P [75].

This potent Ctz-containing Re(I) organometallic compound and four close derivatives were analyzed shortly after by Machado and coworkers using MP-AES for determining Re uptake by *T. cruzi* epimastigotes. Among the five studied [2+1] *fac*-Re(I) tricarbonyl species, complex **61** (Scheme 2) yielded the best results, with an IC_50_ value against *T. cruzi* calculated at 3.43 µM (Table 3). This molecule was selected for the bioanalytical study, which revealed that between 1.16% and 1.28% of rhenium mass was present in the pathogen. The total intracellular concentration of rhenium was independent on the time of incubation (4 h or 24 h), as well as the initial concentration (1 time or 10 times the IC_50_). Subsequent measurements of the organism extracts after exposure to **61** showed that it was mainly located in the soluble proteins fraction, whereas less than 1% of the total Re uptake was detected with DNA or RNA [82]. The same group reported the antitrypanosomal activity of similar complexes one year later. The six Re(I) complexes featuring a Ctz or Ktz ligand were characterized by NMR, IR spectroscopy and MP-AES. Moreover, crystals of compound **62** (Scheme 2) suitable for X-ray diffraction were successfully grown. The coordination species were first tested for their stability in different mixes of DMSO with biological medium, as well as in human plasma using HPLC methods.

**Figure 10:**
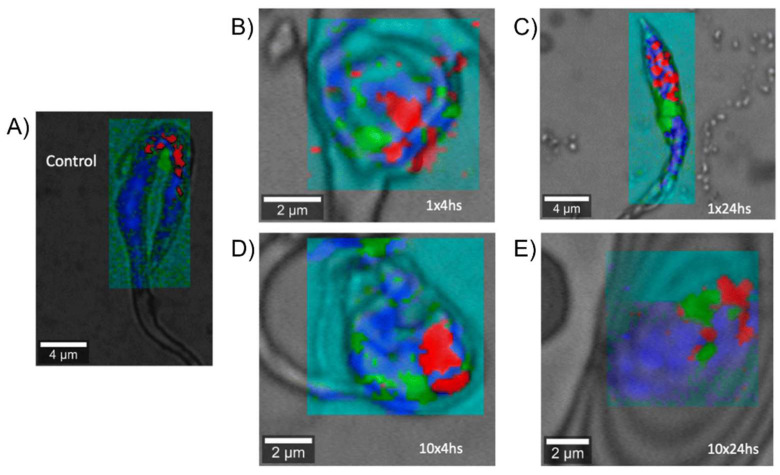
Overlay between optical and confocal Raman images as obtained from selected principal components (red: proteins and DNA, blue: lipids, green: DNA at higher concentrations) analysis of *T. cruzi* treated with (**A**) control, (**B**) **61** at IC_50_ for 4 h, (**C**) **61** at IC_50_ for 24 h, (**D**) **61** at 10 times IC_50_ for 4 h and (**E**) **61** at 10 times IC_50_ for 24 h; obtained and modified with permission [83].

The five Ctz derivatives **61**–**65** (Scheme 2) did not show any sign of decomposition after 24 h, or even more in certain cases. Further HPLC analysis along with the ‘shake flask’ experiment could afford a ranking of each molecule according to its lipophilicity. However, the latter did not necessarily correlate with low IC_50_ values against *T. cruzi* epimastigotes and trypomastigotes. However, it is worth mentioning that all Ctz complexes exhibited better activity than the free AA, and that the most promising results were yielded by compound **61**, which was found to be the most lipophilic one of the series at the same time (Table 3). In addition to a lesser antikinetoplastid potency, some other complexes exhibited a too high toxicity toward Vero cells, which was not the case for **61**. By associating key Raman signals to specific cell components like proteins, DNA and lipids, the authors could then emphasize the important localization disturbance of DNA in parasites treated with **61** compared to the control ones (Figure 10). Therefore, it was concluded that DNA was a potential target of **61**–**65**. This hypothesis was corroborated by UV-Vis titration with ctDNA and by molecular docking calculations both supporting the occurrence of weak interactions with the major groove. Nevertheless, measurements of ergosterol precursors contents in *T. cruzi* epimastigotes treated with **61** revealed a significant accumulation of lanosterol, which possibly implies a mechanism of action identical to Ctz. Furthermore, docking studies toward the C_14_-demethylase enzyme confirmed a better affinity with **61** compared to the free AA [83].

Later that year, Zobi and coworkers carried out a computational study on the same Re(I) tricarbonyl scaffold bearing an axial Ctz, and synthesized such complexes designed with a bipyridine derivative of pinene as bidentate ligand, before assessing their antibacterial activity. Compounds **57**, **66**–**69** (Scheme 2) were characterized by NMR, IR and UV-Vis spectroscopy, as well as by X-ray crystallography in the case of **57**. The latter was first published by Mendes et al. [75], who highlighted a plausible mechanism of action for **57** consisting of the inhibition of the MurG-mediated conversion of Lipid I into Lipid II (Figure 9) [75]. Here, the authors took advantage of this knowledge by performing docking calculations of almost sixty Re(I) coordination species of Ctz toward a homology model of *S. aureus* MurG. The candidates displaying the highest negative binding scores **66**–**69** were synthesized and tested, along with **57**, against a sensitive and a resistant strain of *S. aureus*. Surprisingly, compound **57** displayed the lowest MIC and MBC values, whereas the pinene derivatives were found to be slightly less active (**66**–**68)** or inactive (**69**), despite their superior results in silico (Table 2). Subsequent refined docking studies of the five complexes yielded more similar docking scores than initially calculated, which could explain the comparable MIC values obtained during the biological tests. The authors concluded positively, as their computational screening made it possible to select potent antimicrobial candidates, especially considering that Mn(I) analogs [75] were usually innocuous when the bpy moiety was functionalized in the 4 and 4′ positions [76].

In 2015, Govender and coworkers developed a new Ag(I) complex of Ctz (**70**, Scheme 2) and encapsulated it in solid lipid nanoparticles, in order to monitor its delivery profile in bacteria. The coordination compound was characterized by NMR spectroscopy and HRMS. The viability of Hep-G2 cells exposed to the complex was first assessed, in order to make sure that the displayed toxicity was acceptable. As the cell viability remained above 80%, even at a concentration of 100 µg/mL (125 µM), its activity against methicillin-sensitive and -resistant *S. aureus* was determined. The resulting MIC values were measured at 9.76 µg/mL (22.7 µM wAA) and 15.62 µg/mL (36.34 µM wAA), respectively, while the values of the free AA were both at 31.25 µg/mL (90.62 µM, Table 2). The mechanism of action was briefly addressed by gel electrophoresis of cell wall proteins from *S. aureus* treated with **70**, which showed affinity for proteins in the case of both the methicillin-sensitive and -resistant strains. After encapsulation, the MIC values both increased to 52 µg/mL, which constitutes an improvement in efficiency (7.3 µM wAA) after consideration of the drug loading (4.84%). However, the changes concerning the activity sustainability constituted the most relevant difference. Indeed, the encapsulated compound retained unchanged antibacterial properties even 54 h after administration and up to 72 h against the methicillin-sensitive strain, whereas the free coordination species lost all detectable activity after 18 h [77].

The year after, Colina-Vegas et al. reported the synthesis, cytotoxicity and antimalarial activity of three octahedral Ru(II) complexes coordinated with bpy, Ctz, chloride and a bidentate P-P ligand (**71**–**73**, Scheme 2). The obtained structures were confirmed by NMR, IR, UV-Vis spectroscopy, elemental analysis, ESI-MS, CV and molar conductivity measurements. The anticancer potency of the complexes was assessed against A549, DU145 and MCF7 tumoral cells, as well as the L929 healthy cell line. Even though **71**–**73** displayed lower IC_50_ values than Ctz and, excluding DU145 cells, than cisplatin, most SIs lay below 1 (Table 1). Namely, **72** yielded SIs of 0.14 and 0.28 regarding the DU145 and MCF7 lines, respectively, while all compounds gave more promising results against A549 cells with SIs slightly above 1. The data collected upon in vitro treatment of *M. tuberculosis* with each complex showed a significant increase in activity against this pathogen compared to Ctz, with MIC values measured between 10 µM and 20 µM (Table 2). Additionally, the affinity of **71**–**73** for DNA was determined. For this purpose, the hypochromism effect observed by means of UV-Vis spectroscopy upon titration with ctDNA was exploited to calculate the K_b_ of the complexes. It was found that **73** possesses approximately two-fold superior DNA binding ability than its two analogs, **71** and **72**. This tendency was consistent with subsequent EB displacement experiments, which confirmed that this ferrocene derivative induced the most significant fluorescence quenching response. Despite the lack of results that would allow the establishment of a convincing mode of binding, viscosity measurements, CD spectroscopy, gel electrophoresis analysis and guanosine monophosphate interaction study monitored by NMR spectroscopy could exclude intercalation or other strong binding interactions. Finally, temperature-dependent fluorescence spectroscopy proved a significant affinity for bovine serum albumin (BSA) through hydrophobic interactions [64].

**Figure 11:**
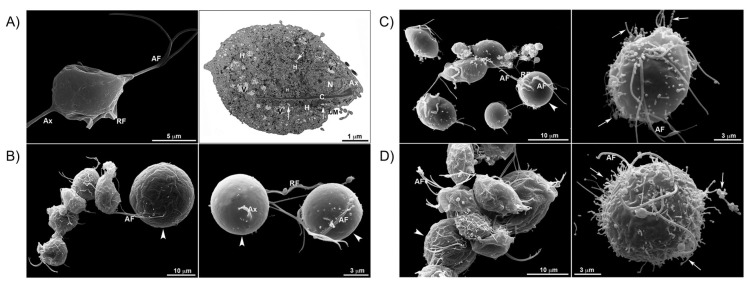
SEM and TEM images of *T. vaginalis* (**A**) untreated, (**B**) treated with Ctz, (**C**) treated with **51** and (**D**) treated with **74**; anterior flagella (AF), recurrent flagella (RF), axostyles (Ax), costa (C), anteriorly positioned nuclei (N) and hydrogenosomes (H) are indicated as well as peripheral vesicles (arrows in **A**), cell rounding (arrowheads) and membrane projections (arrows in **B**, **C** and **D**); obtained and modified with permission [84].

Navarro and coworkers reported the synthesis of two Zn(II) complexes bearing a Ctz ligand (**51** and **74**, Scheme 2) and their activity against *T. vaginalis*. These compounds were analyzed by NMR, IR spectroscopy, elemental analysis and molar conductivity measurements. The IC_50_ value of **74** was measured at 4.9 µM (9.8 µM wAA) against the parasite, lower than that of the free AA (17.2 µM, Table 3). Furthermore, the authors emphasized the necessity of complexation by administrating 2:1 mixtures of Ctz and Zn(ac)_2_ to the pathogen, which yielded an IC_50_ value similar to that of uncoordinated Ctz. SEM and TEM made it possible to visualize the effect of the two compounds on the ultrastructural features of the protozoan, with the most evident one consisting of membrane projections that even detached from the cell body in some cases. These important alterations were observed with growing magnitude from free Ctz to **51** and finally **74**, which induced disorganized Golgi lamellae, as well as altered hydrogenosomes (Figure 11). Indeed, immunofluorescence microscopy further highlighted the impact of the acetate complex **74** on hydrogenosomes morphology.

Despite their antiproliferative property, these organometallic compounds did not induce any significant decrease in pathogen viability at their IC_50_. However, HFF cells stayed viable even at concentrations two and four times higher upon treatment with **51** and **74**, respectively [84]. Along with these derivatives of Ctz, the authors tested ten others Cu(II) and Zn(II) complexes of Ctz and Ktz against several fungal pathogens, namely *Candida albicans*, *Cryptococcus neoformans* and *Sporothrix brasiliensis*. The organometallic compounds were identified by the means of NMR, EPR, IR, UV-Vis spectroscopy, elemental and molar conductivity analysis. Moreover, among the six Ctz complexes, two solid structures (**74** and **75**, Scheme 2) could be determined by X-ray crystallography. All Ctz coordination compounds yielded similar to 8-fold lower MIC_50_ values than that of the free AA, which was determined at 0.5 µM against the three fungi lines (Table 4). However, the best results were displayed by the Zn(II) complexes **51**, **74** and **76** (Scheme 2) [90].

In 2020, Mohamed et al. reported thirteen Ag(I) NHC species derived from Ctz and assessed their lipophilicity and cytotoxicity, as well as their affinity for the cell membrane. The synthesis of the complexes was carried out in mild conditions involving the formation of the NHC precursor salt followed by deprotonation and coordination with Ag_2_O. The compounds were characterized by NMR spectroscopy, HR-MS and elemental analysis. In addition, the X-ray crystal structures of **77**–**81** (Scheme 2) were obtained. The complexes were tested against tumoral Panc10.05, MiaPaCa2, BE and healthy ARPE19 cell lines. Globally, **77**, **82** and **83** (Scheme 2) yielded the highest Sis, ranging from 4.27 to 17.47, against Panc10.05 and MiaPaCa2, i.e., better than cisplatin (Table 1). Interestingly, these compounds were found to be among the less lipophilic of the series, according to log*P* measurements.

The iodide containing molecules **81**, **84** and **85** (Scheme 2) showed results more dependent on the type of cell line, with best SI values against BE cells (7.31, 2.42 and 8.51, respectively). In order to elucidate the SAR of the complexes, the author performed an RCV biomembrane interaction study. It was possible to deduce the beneficial role of the aromatic chloride substituent in the affinity for the sensor. Concerning the *N* substituents of the NHC ligands, the hydroxyethyl and benzyl derivatives exhibited the same positive tendency. Comparing the coordination compounds with their respective NHC precursors, similar results were obtained, although complexation rendered the interaction with the lipid bilayer irreversible. However, the data suggested the disruption of the monolayer instead of its penetration. Overall, moderate correlation between the LOD values and the previously discussed IC_50_ ones could be concluded [65].

Navarro and coworkers recently investigated similar NHC derivatives of Ctz bound to an Ag(I) metal center. Compound **84**, along with compound **86** (Scheme 2) and all the Ktz analogs, were tested for their anticancer properties. Their structure was confirmed by means of NMR, IR, UV-Vis spectroscopy, ESI-MS, elemental analysis and molar conductivity measurements. The cytotoxicity tests against cancerous B16F1 and CT26WT cell lines, as well as s healthy BHK21 cell line, revealed the superior activity of both NHC coordination species of Ctz **84** and **86** compared to the NHC derivative of the AA, even though it displayed particularly high IC_50_ values (Table 1). Additionally, all Sis were calculated at 1 or above. Concerning the biological targets of the complexes, no interaction with DNA could be observed by UV-Vis titration, viscosity measurements or gel electrophoresis. However, fluorimetric titration of BSA revealed the affinity of **84** and **86** for this macromolecule, notably **86**, which exhibited a K_b_ value above 10^6^ M^−1^ [66].

In the previous section, the study of Aziz et al. [88], evaluating the affinity of three Cr(III) complexes for DNA, was already discussed. Upon UV-Vis titration, the Ctz derivative considered in this work exhibited a 10-fold higher K_b_ (10^7^ M^−1^) for ctDNA, compared to the two other coordination compounds [88]. In a similar publication by Hussien and coworkers on V(IV) coordination species of AAs, the Ctz-containing molecule **87** (Scheme 2) gave less promising results compared to its already discussed Mcz analog **20**. Indeed, the former yielded a three times lower K_b_ value toward ctDNA, and its IC_50_ values were measured at 8.27 µM against HepG2 cells and 6.97 µM against MCF7 cells (Table 1) [57].

As mentioned above, Stevanovic et al. [74] investigated the improvement of AAs biological activity upon complexation to a Au(III) metallic core. Similar to the inorganic derivatives of Ecz **25** and Tcz **26**, compound **29** showed a decrease in MIC magnitude varying from 1.5- to 7-fold against *Candida* species compared to Ctz (Table 4). A notable exception concerned *C. albicans*, against which the opposite phenomenon was observed, meaning a 15-fold increase in MIC value after coordination. Against bacteria, **29** displayed comparable results to **25** and **26** as well. The lowest MIC value was measured against *S. aureus* at 9.6 µM (Table 2) [74].

A recent work on the coordination derivatives of Bfz and Ctz was reported by Busto and coworkers who investigated the anticancer properties of three chloro Pt(II) complexes bearing a bidentate 2-phenylpyridinate chelate and a monodentate imidazolyl-based ligand. The characterization was carried out by NMR, IR, UV-VIS spectroscopy, HR-MS and elemental analysis. Additionally, X-ray crystallography provided the 3D structures of compounds **88** and **89** (Scheme 2). In subsequent cytotoxicity studies against SW480, A549 and A2780 cell lines, **89** yielded IC_50_ values of 38.3 µM, 19.0 µM and 6.1 µM, respectively, which is lower than that of cisplatin in every case (Table 1). The Ctz coordination compound **88** displayed less promising results, with IC_50_ values between 50.6 µM and 68.7 µM. Conversely, ICP-MS revealed a very poor cellular uptake of complex **89** compared to **88**. These confounding data could be explained by assessment of BSA and plasmid DNA binding ability through gel electrophoresis. The obtained patterns suggested a significantly higher affinity toward BSA for **89** than for **88**, which potentially implies that the former could undergo sequestration in the blood medium. Meanwhile, both species were found to affect plasmid DNA migration. CD spectroscopy confirmed these outcomes and afforded evidence toward a covalent binding mode to DNA. Despite such a conclusion, the reasons for the superior activity of the Bfz derivative **89** remained unclear and, therefore, the author explored the ROS production possibly induced by the two molecules in A549 cells by fluorescence microscopy. Compound **89** was found to be by far the most efficient ROS producer. This feature was supported by mitochondrial membrane potential measurement. Indeed, the observed decrease constituted a relevant indication of ROS presence, whereas **88** exhibited no such impact. A similar dichotomy arose in the case of cell cycle analysis by flow cytometry, as exposure to **89** resulted in higher cell population in G_0_/G_1_ phase at the expense of the one in S phase, whereas its Ctz analog **88** showed no effect. Finally, Annexin V staining suggested different apoptosis trigger mechanisms for **88** and **89**, that is a similar percentage of cells in necrosis or late apoptosis for the former and a higher number of cells in early apoptosis for the latter [67].

### 2.3. Coordination Compounds of the Ktz Family of AAs

Almost a decade after the advent of Mcz and Ctz, Janssen patented Ktz and Itz, the two AA being approved for medical use four and ten years following their discovery, respectively [48]. In these newer drugs, the main scaffold of Mcz connecting the azole ring to a bischlorinated phenyl group remains identical (Scheme 1). However, this skeleton is linked through a dioxalane ring to a massive side chain. The latter was designed to increase oral bioavailability [111] and consists of a phenol ether attached in *para* position to an *N*-substituted piperazine. Apart from the tail of this sequence varying from Ktz to Itz, the more subtle substitution of imz for triazole induced a biologically critical difference. Indeed, this innovation was intended to increase the selectivity for fungal CYP and, therefore, lessen the possible side effects by undesirable affinity with the corresponding human anit-target [11]. Conversely, the higher Lewis basicity of the traditional imz group allows easier coordination to a metal center, which could explain the superior popularity of Ktz in the literature discussed below.

The group of Sanchez-Delgado produced the first publication related to the coordination chemistry of Ktz. As mentioned in the previous section, this antitrypanosomal study aimed at comparing three Ru(II) and Ru(III) complexes with compound **27**. The two Ktz derivatives (**90** and **91**, Scheme 2) yielded a more than 3.5-fold inhibition percentage increase compared to the free AA against *T. cruzi*. This improvement was significantly better than the one observed for the Ctz analog **30**, although **27** still exhibited the best results [100]. The same authors published a very similar work on Au(I) and Cu(II) coordination compounds. The Ktz species investigated displayed almost identical *T. cruzi* growth inhibition potency as the already discussed Ctz complexes. However, while Ctz alone showed no activity against the parasite, uncoordinated Ktz did. As a consequence, it is somewhat surprising that the metallic derivatives of the latter did not yield superior results [101].

The investigation of compound **91** as a potential anticancer agent by Rieber and coworkers was performed at the same time as its Ctz analog **27**. Although the species exhibited comparable antiproliferative results against C8161 melanoma monolayers, the ability of **91** to fragmentize PARP was significantly superior. Furthermore, the same complex induced caspase-3 activation in C8161 and HT29 cells, unlike **27**, and that is why it was selected for further cytotoxicity assessment. Firstly, quantitation of PARP fragmentation by laser scanning cytometry revealed that this biological phenomenon was occurring almost independently of the mutation of the p53 genes, thus indicating the potential of the compound to treat resistant strains. Subsequently, **91** was shown to importantly decrease cell population in S + G_2_, again in wild types p53 as well as mutated ones. The capacity of this promising agent to release mitochondrial components and, compared to cisplatin, its superiority with respect to PARP fragmentation and triggering of pre-apoptotic signals were confirmed. Finally, the authors tested **91** on C8161 spheroids, a more recalcitrant tumoral form on which most cytotoxic power was retained. However, only its simultaneous exposure with a blocking antibody could yield positive results against A431 spheroids possessing mutated p53 [103].

The library of compounds analyzed in the last cited publications was complemented and tested against *S. cerevisiae* by Navarro and coworkers. These Ktz derivatives were either previously published or not characterized. Their MIC values lay in the low micromolar range, close to that of the uncoordinated AA at 1 µM. All measured IC_50_ values on human neutrophils were several times higher than the corresponding MICs, and even though **91** seemed to significantly improve the defense ability of these white blood cells regarding phagocytosis and killing percentages, such results are hard to put in perspective, due to absence of reference data in the paper [104]. In 2018, the authors continued their antifungal study on these types of Au(I), Cu(I) and Pt(II) complexes bearing AAs, with compounds featuring Ktz ligands. Compared to the data obtained with the Ctz-containing analogs, there is almost no relevant variation to emphasize concerning the biological activity, apart from the general absence of MDS, probably due to the high activity of uncoordinated Ktz. Exceptions were mainly observed in the case of the two phosphine-containing compounds (**92** and **93**, Scheme 2), again proving that the presence of such a ligand was essential for better fungicidal properties (Table 4) [89].

In 2013, the group of Sanchez-Delgado opened the biological investigation into Ru(II) piano stool complexes by reporting the activity of six Ru(II) compounds bearing a Ktz moiety against *L. major* and *T. cruzi*. Among the species, two displayed an octahedral geometry and the four others the popular piano stool structure with a cym ligand. These structures were confirmed by NMR spectroscopy and elemental analysis, as well as X-ray crystallography in the case of complex **94** (Scheme 2). The latter yielded the best antiparasitic results, as it showed the highest increase (more than 2.4 times) in activity upon Ktz coordination against *L. major* promastigotes and was the only species to show any activity, although modest, against *T. cruzi* epimastigotes (Table 3). The corresponding LD_50_ values were calculated at ca. 1 µM, and even against the intracellular amastigote form of *T. cruzi*, 50% inhibition was measured after exposure to a 0.1 µM concentration of **94**.

The same compound exhibited no significant toxicity toward Hs27, U2OS, RAW264.7, IPϕ and mouse macrophage mammalian cells. In order to rationalize the superior biological properties of **94** over the other compounds of the series, the authors highlighted that analog data were obtained in their previous work on Ctz [80]. Indeed, **37** possessed the most promising features with respect to the other derivatives. An important disparity between **94** and the three remaining piano stool species (**95**–**97**, Scheme 2) consists of the presence or absence of a non-labile bidentate ligand. This fact seems relevant, as **95**–**97** cannot easily undergo aquation in the physiological medium and, therefore, do not form the hypothetically active compound. Such a reaction was proven—by NMR, UV-Vis and molar conductivity analysis—to occur rapidly in aqueous DMSO for the chlorinated piano stool derivative [85]. The same series of Ru(II) Ktz compounds was analyzed as part of a fluorimetric titration study assessing binding ability to HSA and apotransferrin. For both proteins, the obtained data were similar. In particular, **95**, **97** and **98** (Scheme 2) yielded K_b_s above 10^8^ M^−1^, while the corresponding value for the other complexes were several orders of magnitude lower [112].

The same library was investigated again in an earlier discussed antineoplastic work of the authors, along with the Ctz analogs. Globally, the obtained cytotoxicity data strongly depended on the compound and the cell line, so that no relevant tendency could be highlighted (Table 1). However, the SIs calculated with respect to HaCaT cells lay far above 1, with scarce exceptions. The cytometric analysis assessing cell death mechanism yielded confounding results as well. Indeed, while certain complexes presented no significant inclination toward apoptosis or necrosis, the former pathway was clearly preferred upon treatment of YT cells with **95**, **96** and **98**. Concerning the DNA affinity study by CD spectroscopy and melting point measurements, the Ktz coordination compounds displayed the same properties as the Ctz ones, that is, a covalent groove binding ability [60].

Introducing a triphenylphosphine ligand onto such a piano stool Ru(II) backbone, the group of Navarro published an already mentioned anticancer study on **99** (Scheme 2). While yielding K_b_ values toward HSA and binding properties with DNA similar to its Ctz counterpart **42**, this compound exhibited the lowest quenching ability during the Hoechst 33,258 competitive ctDNA affinity test. The amount of metal attached to DNA was measured at only 75 nM/mg or 1 Ru atom per 20 DNA base by ICP-OES, which confirmed its non-covalent binding mode. Against tumoral and non-tumoral cell lines, **99** showed almost identical IC_50_ values compared to **42**, whereas free Ktz possessed three times lower cytotoxicity than Ctz toward most tested lines (Table 1). As a consequence, **99** afforded the best example of MDS, although it was not selected for subsequent microscopy assays [62]. The same complex **99** was tested for its antileishmanial properties shortly after. In the assay, it yielded the lowest IC_50_ values of the series, i.e., at 0.15 µM and 0.01553 µM against *L. amazonensis* promastigotes and intracellular amastigotes, respectively (Table 3). With respect to its low toxicity toward murine macrophages and the morphological alterations it induced on the promastigote form of the parasite, **99** exhibited the same promising results as its parental species **42 [81]**. The quality of these works on Ru(II) piano stool complexes was questioned by Gasser and coworkers, more specifically concerning **94**. Indeed, this complex partially degraded in DMSO, like its Mcz **10**, Tcz **13** and Ctz **37** counterparts. As a consequence, the obtained IC_50_ values against HeLa, L6 cell lines and *T. cruzi* were similar when exposed to the molecule or a mixture of [Ru(cym)(DMSO)Cl_2_] with Ktz. The dissociation of the AA was further emphasized by a comparative NMR study in DMSO-d6, which contradicted the publication of Iniguez et al. [85], where the authors stated that no release of Ktz was detected in aqueous DMSO, but rather the formation of the aqua coordination compound by chloride dissociation [71].

In their previously discussed publication on Mcz, Ctz and Ktz derivatives designed with a Mn(I) metallic core, Simpson et al. [73] tested compounds **100** and **101** (Scheme 2) against various pathogens. The species showed less promising results overall against G+ and G− bacteria (Table 2), as well as against *T. brucei* (Table 3) regarding the other compounds of the series (**16**, **17** and **55**). However, the two complexes, especially **100**, displayed a considerable increase in activity compared to uncoordinated Ktz, as well as low toxicity toward mammalian cells [73]. A similar tricarbonyl complex featuring a Re(I) core and a Ktz ligand was reported by Soba et al. [83], along with five already discussed Ctz-containing compounds. The Ktz derivative **102** (Scheme 2) displayed less promising results compared to the latter, as it degraded in less than 24 h in DMSO:fetal bovine serum 1:1, as well as in human plasma. This instability observed in certain biological media could partially explain the innocuous nature of **102** toward *T. cruzi* trypomastigotes, which lead the authors to discard this molecule for further analysis. [83]

The first complexes of Itz investigated for antimicrobial purposes were reported in 2020 by de Azevedo-França et al. [86] Featuring a Zn(II) metal center, compounds **103** and **104** (Scheme 2) were characterized by NMR, IR, UV-Vis spectroscopy, ESI-MS, elemental and molar conductivity analysis. Moreover, their activity, along with that of the uncoordinated AA, against *L. amazonensis*, *T. gondii*, *T. cruzi*, *S. brasiliensis* and *S. schenkii* was assessed in addition to their toxicity toward healthy RAW264.7, HFF, LLCMK_2_ and red blood cells. Apart from scarce exceptions, both compounds displayed promising antikinetoplastid activity with IC_50_ values in the low nanomolar range, i.e., significantly lower than Itz, and negligible toxicity (Table 3). The antifungal results emphasized the synergistic effect of complexation as well. Indeed, while the MIC values of the uncoordinated AA were measured at 0.6 µM and 1.25 µM against *S. brasiliensis* and *S. schenkii*, respectively, the complexes were up to six times more active (Table 4). Further data were provided by SEM and TEM study of ultrastructural impact on *L. amazonensis* promastigotes and *T. cruzi* epimastigotes upon exposure to **103** and **104**. These analysis revealed cell rounding, strong mitochondrial swelling, presence of vacuoles in the cytoplasm or in some organelles and, in the case of *T. cruzi*, various flagella alterations [86].

**Figure 12:**
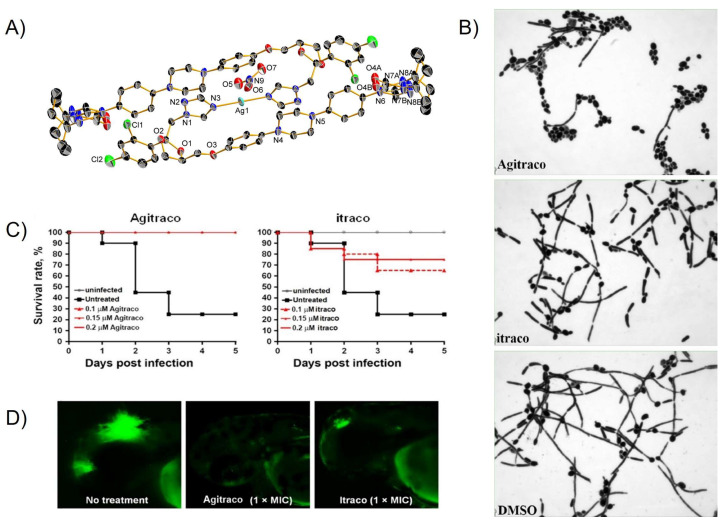
(**A**) Crystal structure of **105** with thermal ellipsoids drawn at the 50% probability level. (**B**) Microscopy images of hyphae formation in *C. albicans* untreated and treated with Itz or **105**. (**C**) Kaplan–Meier survival curve of zebrafish embryos non-infected, infected by *C. albicans* or infected by *C. albicans* and treated with different concentrations of **105** (**left**) and Itz (**right**). (**D**) Fluorescence microscopy images of zebrafish embryos infected with fluorescently labeled *C. albicans* cells (green), untreated and treated with **105** or Itz at their respective MICs; obtained and modified with permission [91].

Pavic and coworkers produced another work on Itz coordination chemistry with the synthesis of a Ag(I) complex bearing this AA (**105**, Scheme 2) and assessment of its anticandidal activity in vitro as well as in vivo. The X-ray structure of **105** was, at that time, the first reported one of an Itz metallic derivative (Figure 12A). The species was further characterized by NMR, IR, UV-Vis spectroscopy, elemental and molar conductivity analysis. It yielded MIC values below 0.4 µM (0.8 µM wAA), that is, lower than Itz against *C. albicans*, *C. glabrata* and *C. parapsilosis* (Table 4)*,* as well as an IC_50_ and a LC_50_ value both slightly higher toward healthy MRC5 cells and zebrafish, respectively. The authors partly attributed this substantial increase in activity to the superior capacity of **105** to produce ROS compared to the uncoordinated AA, as proven by flow cytometry using a fluorescent probe. Moreover, the ability of this complex to almost completely inhibit the transition to hyphae form was confirmed by microscopy (Figure 12B). These promising preliminary results lead to subsequent in vivo assays in which **105** saved all zebrafish infected with *C. albicans* at half its in vitro MIC value (Figure 12C). The biological effect of the coordination compound was followed by fluorescence microscopy of infected zebrafish embryos (Figure 12D). Finally, **105** was shown to bind BSA by fluorimetric titration with a K_b_ value slightly above 10^3^ M^−1^ [91].

**Scheme 3:**
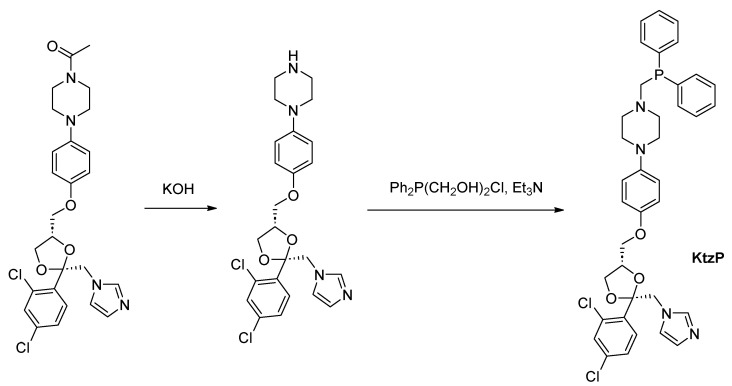
Synthesis of KtzP [113].

Starosta and coworkers published the luminescent and antifungal properties of two Cu(I) complexes bearing a Ktz-like moiety (KtzP, Scheme 3). The coordination was designed to occur via a phosphine group attached to the drug scaffold by a modified Mannich condensation [113], and not by the usual *N*3 of the imz ring. The structures of the resulting complexes (**106** and **107**, Scheme 2) were verified by NMR spectroscopy, elemental analysis, as well as single crystal X-ray diffraction for **106**. After confirmation of MLCT phosphorescence in aqueous media, both species were tested against *C. albicans*. The selected strains included clinical isolates with Fcz resistance through efflux pump overexpression. However, the obtained IC_50_ values remained in the nanomolar range, lying between 0.39 µM against the sensitive clinical isolate and 0.78 µM against the resistant one with overexpression of CDR1 and CDR2 (Table 4). These data corresponded to an increase in activity between 7-fold and 60-fold compared to KtzP. Additionally, no significant toxicity toward healthy NHDF cells was measured upon treatment with **106**. Such an improvement in drug efficiency suggested a distinct mechanism of action and indeed, molecular docking studies revealed a poor capacity to access the binding cavity of *C. albicans* CYP51 for both Cu(I) derivatives. The luminescent features of **106** and **107** granted additional evidence, showing the accumulation of these complexes in *C. albicans* vacuoles without any observable membrane damage, which is usually provoked by AAs [92].

The impressive library of twelve Cu(II) and Zn(II) complexes bearing Ctz or Ktz reported by Navarro and coworkers comprised six novel Ktz derivatives, which were tested against fungal pathogens as well. These species exhibited overall better results than their Ctz analogs. In particular, compounds **108** and **109** (Scheme 2) yielded MIC_50_ values globally below 0.015 µM (0.030 µM wAA) against *C. albicans*, *C. neoformans* and *S. brasiliensis*, whereas MIC_50_ values of the free AA were 0.25 µM, 0.06 µM and 0.03 µM, respectively (Table 4). Conversely, the complexes displayed higher toxicity toward healthy LLCMK_2_ cells compared to Ktz, although the corresponding CC_50_ values, measured in the micromolar range, still resulted in excellent SIs. **108** and **109** were selected for additional biological analysis by SEM, which revealed important shape modifications in *S. brasiliensis* compared to treatment with Ktz alone [90].

In 2023, the study of Navarro and coworkers, discussed above, on Ag(I) complexes featuring NHCs based on AA scaffolds and their anticancer properties included three Ktz derivatives. They displayed better results than their Ctz analogs, especially **110** and **111**. Indeed, their IC_50_ values were globally measured lower than the ones of the Ktz NHC derivative, whereas the latter, unlike in the case of Ctz, showed improved cytotoxicity compared to the unfunctionalized AA (Table 1). Furthermore, **110** and **111** yielded SIs around 10 against B16F1 cells. These two coordination species were among the best BSA binders as well, with K_b_ values both calculated above 10^6^ M^−1^ during fluorimetric titration analysis [66].

### 2.4. Coordination Compounds of the Fcz Family of AAs

Pfizer patented Fcz and Vrz more than ten and twenty years, respectively, after the development of the first AAs. Both were medically validated several years after their discovery, i.e., in 1988 for Fcz and in 2002 for Vrz [48]. The scaffold of these newer drugs can rationally be considered as a derivative of Mcz, where the ether functionality was hydrolyzed, the two remaining chlorides were replaced by fluorides, the original imz moiety was changed for a triazole ring and an additional methylheteroaryl substituent was implemented on the benzylic carbon (Scheme 1). In particular, the latter innovation allows the presence of a second coordination site through the introduction of an additional nitrogen-containing aromatic group. However, the interaction of Fcz and Vrz with various transition metals was investigated mainly in the field of coordination polymers and MOFs, with no particular interest in biological applications [114,115,116,117,118,119,120,121,122,123,124,125,126,127,128,129,130,131,132,133,134,135,136]. Exceptions however exist and will be discussed in the following paragraphs.

In the first publication involving the coordination chemistry of Fcz, Lupetti et al. [137] reported the radiolabeling of this AA using ^99m^Tc, with the purpose of fungal infection imaging. Preliminary in vitro assays showed higher binding ability of the Fcz derivative toward *C. albicans* and, to a lesser extent, *A. fumigatus* compared to bacterial organisms or activated leukocytes. These results were corroborated in a subsequent in vivo study, where scintigraphic images afforded the biodistribution of the molecule in mice infected by *C. albicans*. Indeed, the radioactive complex accumulated mainly at the infection site with a target-to-nontarget ratio of around 3—at least two times higher than the one measured for sterile inflammations. The compound yielded far less promising data against *A. fumigatus*, which suggested its preference for *C. albicans*. Such selectivity constitutes a desirable property which, with the observed absence of accumulation in the mice liver, showed the potential of this Fcz species in the field of diagnostic imaging [137].

**Figure 13:**
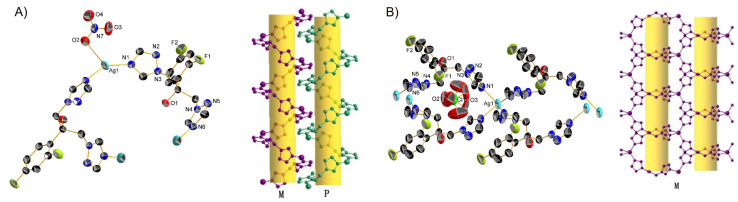
(**A**) ORTEP view of the coordination environment of Ag(I) (**left**) and schematic view of the helical structure (**right**) in **112**. (**B**) ORTEP view of the coordination environment of Ag(I) (**left**) and schematic view of the 2D layered structure with helical arrangement (**right**) in **113**; obtained and modified with permission [93].

Five years later, Zhang and coworkers synthesized two Ag(I) coordination polymers of Fcz and assessed their antifungal potency. The compounds (**112** and **113**, Scheme 2) were characterized by FT-IR and X-ray spectroscopy. Compared with the uncoordinated AA, they exhibited lower (*C. albicans*, *M. mucedo*, *R. tolonifer*) or much lower (*S. cerevisiae*, *P. uniculosum*, *A. niger*) MIC_80_ values (Table 4). The authors hypothesized two possible mechanisms of action, either binding to macromolecules due to the helical structure of both species (Figure 13) or by decomposition of the complexes into active silver salts in the biological medium [93].

Similar Ag(I) coordination polymers designed with Vrz were tested against several fungal pathogens by the same group. Compounds **114** and **115** (Scheme 2) were characterized by NMR and IR spectroscopy, as well as by X-ray single crystal and powder diffraction. Concerning the biological assays, the MIC_90_ values were measured between 0.0625 µg/mL (0.138 µM wAA) and 2 µg/mL (5 µM wAA, Table 4). The activity displayed by **114** and **115** was better than that of the corresponding Ag(I) salts against all selected fungal lines, whereas it was slightly better to slightly worse than uncoordinated Vrz in general. An important exception was *C. albicans*, against which both coordination polymers showed great improvement compared to the free AA (Table 4). The corresponding fractional inhibitory concentration indexes were calculated slightly above or under 0.4, which indicated MDS and demonstrated the capacity of drug complexation to overcome resistance. This phenomenon was explained by PCR analysis of the parasites, where the ability of the molecules to down-regulate the expression of CDR1 gene responsible for encoding an efflux transporter was observed, disrupting the resistance mechanism. Finally, the authors emphasized the selectivity of the two Vrz metallic species by showing their innocuous nature toward HaCaT cells, even at around six times their MIC_90_ value [50].

**Scheme 4:**
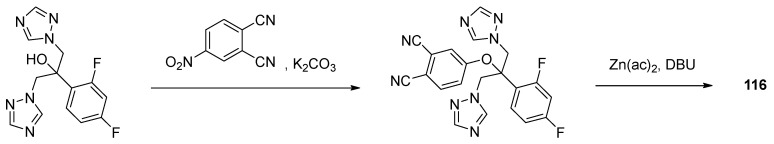
Synthesis of **116** [138].

Durantini and coworkers designed a Zn(II) phthalocyanine complex functionalized with Fcz moieties and tested it for fungicidal photodynamic applications. Compound **116** (Scheme 2) was obtained as a mixture of regioisomers by *ipso* substitution initiated by Fcz on 4-nitrophthalonitrile in basic conditions, followed by tetramerization around the Zn(II) metal center (Scheme 4). The final product was confirmed by FT-IR, FAB-MS and elemental analysis. Additionally, the solubility properties of **116** were studied by UV-Vis and fluorescence spectroscopy, taking advantage of the quenching phenomenon occurring in most solvents because of aggregates formation. Monomerization was found to take place in slightly acidic solution, probably through electrostatic repulsion between individual molecules caused by protonation of the triazole rings, which possess higher basicity than the bridging nitrogen atoms. As a consequence, **116** was dissolved in acidic solutions with the aim to assess its ability to produce ROS. The obtained quantum yield is reported at 0.23 in DMF/H_2_O and 0.30 in reverse micellar systems. Moreover, **116** showed similar activity at 8-fold lower concentration compared to the free AA in an in vitro study on *C. albicans* in the dark (Table 4). Expectedly, the antifungal effect increased upon irradiation. By contrast, the unfunctionalized analog bearing no Fcz substituent exhibited inferior results, possibly because of its lower capacity to interact with *C. albicans* cells, as proven by fluorescence spectroscopy of the fungus extracts [138].

Jezowska-Bojczuk and coworkers reported the anticandidal activity of an equimolar Cu(II)-Fcz system. From such solutions, two different X-ray structures were reported in an earlier publication, where the same authors analyzed mixtures of Cu(NO_3_)_2_ and Fcz at different pH values [139]. According to this work, only complex **117** (Scheme 2) should be formed at pH 7. However, CuCl_2_ was used here instead of Cu(NO_3_)_2_ and without any new characterization attempt, the product was assumed to be a similar binuclear coordination compound. The antiproliferative potency of this molecule was assessed on fifty *C. Albicans* and *C. glabrata* strains. Globally, the species gave slightly inferior (<2-fold increase in MIC) to slightly more promising (<2-fold decrease in MIC) results than the uncoordinated AA [140].

In 2017, Zhao et al. [141] synthesized a complex of Vrz via coordination to a Zn(II) metallic core and analyzed this molecule for its antifungal ability. Compound **118** (Scheme 2) was confirmed by FT-IR, UV-Vis, fluorescence spectroscopy, TGA and elemental analysis. It was extensively tested against eight different species of fungi from the *Candida*, *Cryptococcus* and *Aspergillus* genus, and almost systematically yielded a lower IC_50_ value than that of the free AA (Table 4). The most striking improvement in activity was observed against *A. niger* and *A. flavus* with, in both cases, an IC_50_ value of 0.05 µg/mL (0.1 µM wAA) for **118** and 0.2 µg/mL (0.6 µM) for Vrz [141]. Shortly after, the same authors expanded Vrz coordination chemistry by reporting the fungicidal properties of **119–121**. Compared to the previous work, the change of metal and anion in the salt used for the synthesis significantly impacted the structures of the molecules, as confirmed by IR, UV-Vis spectroscopy, X-ray single crystal and powder diffraction, elemental analysis and TGA. Indeed, in the two coordination polymers obtained from Cu(II) nitrate and sulfate, Vrz exhibited a bidentate coordination mode identical to Fcz that enabled 1D expansion. The same eight fungal organisms were selected for the biological assays, in which the Cu(II) compounds **119**–**121** gave similar results to those of **118** (Table 4). In particular, **119** displayed the best antifungal activity, especially against *C. neoformans*, *A. niger* and *A. flavus* [94].

Surprisingly, analog studies on Cu(II) and Zn(II) coordination compounds of Fcz appeared later in the literature, despite the earlier discovery of this AA. In this field, Qi and coworkers reported a new Zn(II) complex of Fcz featuring a polyoxovanadate anion and tested its anticandidal ability. Even though the structure of compound **122** (Scheme 2) was determined by FT-IR and X-ray crystallography as [Zn_3_(Fcz)_6_(H_2_O)_6_](V_10_O_28_)∙4H_2_O, it is described as “Zn_3_(Fcz)_6_(V_10_O_28_)∙10H_2_O” in the whole paper, which we consider somewhat misleading. The antifungal properties of this species against nineteen strains of the *Candida* genus were assessed. Compound **122** showed superior activity compared to the uncoordinated AA in most cases (Table 4). In particular, the complex yielded more than five-fold lower IC_80_ and IC_50_ values against resistant *C. albicans* HL973 and HL3968 strains. The former was selected for more detailed biological analysis, which revealed the higher antiproliferative capacity of **122** compared to Fcz. For example, the OD_600_ value obtained upon exposure to 8 µg/mL (20 µM wAA) of the complex stayed lower than the one obtained upon treatment with 18.56 µg/mL (60.60 µM) of Fcz. Fluorescence microscopy with acridine orange (AO)/EB staining emphasized the higher antifungal potency of **122** and HPLC analysis of the pathogenic fungus showed abnormally low ergosterol content, even with respect to Fcz treatment, which afforded evidence toward a same mechanism of action compared to the free AA. This hypothesis was reinforced by the upregulation of ergosterol biosynthesis-associated genes, an expected response that was observed by PCR [95].

Concerning research on coordination polymers possessing similar metal centers, Stevanovic et al. [96] published the synthesis, antifungal and antibacterial properties of such Cu(II) and Zn(II) compounds. Compounds **123** and **124** (Scheme 2) were characterized by NMR, IR, UV-Vis, X-ray spectroscopy, ESI-MS and molar conductivity analysis. Toward fungal parasites, both generally yielded better activity than Fcz (Table 4). However, the MIC values of **124** ranged between 1.05 µM (2.09 µM wAA) against *C. parapsilosis* and 3.71 µM (7.42 µM wAA) against *C. krusei*, i.e., up to 16 times lower than that of **123** and 5 times lower than that of the uncoordinated AA. However, the three species (**123**, **124** and Fcz) were determined as potent inhibitors of the yeast-to-hyphae transition on solid Spider medium, whereas fluorescence microscopy revealed **124** as a significantly more efficient antibiofilm agent and **123** as the only compound able to hinder pathogen adhesion onto A549 cells. Additionally, **123** induced the most important ergosterol concentration depletion, as monitored by UV-Vis spectroscopy. In parallel, the authors performed a molecular docking study that exhibited the higher binding affinity of **124** for CYP51.

The contradictory nature of these data could be explained by a divergence in the mechanism of action related to the biological effects of **123**, **124** and Fcz. This hypothesis was further emphasized by the slight antibacterial features only detected in the instance of exposure to **124**. The biological activity consisted in decrease in pyocyanin biosynthesis and biofilm formation in *P. aeruginosa* [96]. Six closely related Cu(II) and Zn(II) coordination polymers of Fcz as well as their antitrypanosomal activity were recently reported by Navarro and coworkers. The compounds were characterized by NMR, IR, EPR spectroscopy, ESI-MS, elemental and molar conductivity analysis. Moreover, the crystal structures of **123**–**126** (Scheme 2) were measured by X-ray diffraction. All molecules were tested against the epimastigote, amastigote and trypomastigote forms of *T. cruzi*. With the exception of **126**, the coordination species always yielded lower IC_50_ values against promastigotes and amastigotes than Fcz alone with excellent SIs calculated toward HFFL cells, in which the amastigotes were cultivated (Table 3).

Indeed, only **123** and **125** showed a lower than 25 SI, probably because of increased cytotoxicity due to Cu(II) poisoning. The only polymer able to significantly induce lysis on the trypomastigote strain was compound **124**. For this reason, and because it exhibited the best antiparasitic results against epimastigotes and amastigotes, this compound was selected to observe the morphological damages it induced on *T. cruzi* by SEM and TEM. Microscopy pictures revealed that the epimastigote form experienced important shape alteration, disorganization of the Golgi apparatus and formation of vacuoles in the cytoplasm upon exposure to **124** [87].

In 2017, Ali et al. [142] published six coordination compounds of Fcz and assessed their fungistatic properties. The complexes featured a Cd(II), Co(II), Cu(II), Fe(II), Mn(II) or Ni(II) metal center, as well as two chloride ligands and various hydration degrees. They were characterized by NMR, IR, UV-Vis spectroscopy, molar conductivity measurement, elemental and morphological analysis using SEM. Concerning the biological results, the authors assessed *A. niger* and *C. albicans* growth inhibition at fixed concentration, which revealed the innocuous nature of all molecules as well as Fcz toward *A. niger*. On the *C. albicans* strain, the uncoordinated AA surprisingly exhibited better activity than its metallic derivatives [142]. A few years later, further investigation on these coordination compounds of Fcz was carried out by means of X-ray powder diffraction, TGA, DSC and anticancer analysis. While the free AA was found to be inactive against the four tested tumoral cell lines (SNB19, HCT15, COLO205, KB31), only the Cu(II) derivative **123** yielded promising results, with IC_50_ values between 13.04 µM and 60.90 µM (Table 1) [68].

Navarro and coworkers published a study on the macromolecules affinity and cytotoxicity of four Ru(II) organometallic compounds bearing an AA ligand. Only the previously discussed Ctz complex was extensively analyzed, biologically speaking. The remaining species, among which were compounds **129** and **130** (Scheme 2), were tested for their HSA, as well as their DNA binding ability and anticancer activity. These two Fcz derivatives yielded K_b_ values comparable to the ones of the Ctz- **42** and Ktz-containing **99** molecules, apart from **130**, whose affinity with HSA could not be detected. Furthermore, the same binuclear complex showed IC_50_ values above the considered concentration range against each of the five cell lines. However, **129** exhibited an overall slightly lesser cytotoxicity than **42** and **99**, with undesirably lowest IC_50_ values against the two healthy cell lines (Table 1). However, these data were still found promising, considering the innocuous nature of Fcz [62]. The same authors discussed the chemistry and antileshmanial activity of these four Ru(II) complexes in a later work. In particular, the crystal structure of **130** was elucidated by X-ray diffraction. Unfortunately, this compound and compound **129** were the only ones of the series to display no antiproliferative effect on *L. amazonensis*, and were thus discarded from further biological analysis [81].

In the present review, the only discussed study featuring MOF chemistry was published by Su et al. [51], who examined the activity of two Vrz-containing Zn MOFs against fungal biofilms. The purpose of this work was to load the AA into the well-established framework derived from 2-methylimidazole-Zn salt (ZIF-8), which was achieved by complexation of Vrz onto the Zn(II) ion followed by addition of 2-methylimidazole. The resulting Vrz-inbuilt ZIF-8s synthesized with 40 mg and 80 mg (**131** and **132**, respectively, Scheme 2) of the AA per 200 mg Zn(NO_3_)_2_ and 2 g 2-methylimidazole were characterized by DLS, zeta potential measurements, SEM, TEM, X-ray powder diffraction, TGA and BET analysis. Furthermore, TEM was performed to quantify the pH dependent drug release, which followed a zero-order kinetics and reached a plateau after 30 h at pH 7.4 and 12 h at pH 5.0. This acidity responsiveness was observable during biological assays against *C. albicans* as well.

**Figure 14:**
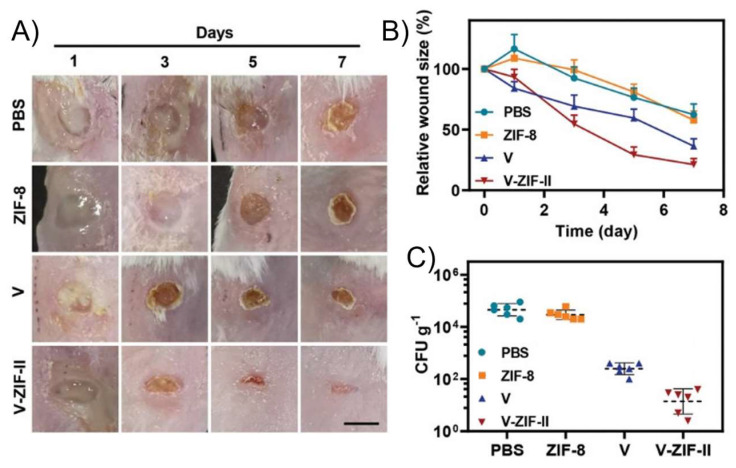
(**A**) Pictures of the wound at different times of the treatment with PBS, ZIF-8, Vrz and **132**. (**B**) Graph of wound size relative to the wound size at day 0 during the course of treatment with PBS, ZIF-8, Vrz and **132**. (**C**) Graph of CFU in wound tissue after seven days treatment with PBS, ZIF-8, Vrz and **132**; obtained and modified with permission [51].

Indeed, while both **131** and **132** exhibited worse results than the uncoordinated AA at physiological pH, their MIC (0.1 µg/mL) and MFC (0.39 µg/mL) values were identical and lower, respectively, than Vrz at pH 5.0. After taking into account the drug loading and converting these data into micromolar, the superior antifungal properties of **131** and **132** become even more evident (Table 4). Considering that the mode of action of the free or MOF-inbuilt drug was similar, as corroborated by SEM and AO/EB permeability fluorescence analysis, the authors hypothesized that **131** and **132** showed slightly better potency at damaging the fungal membrane thanks to electrostatic affinity with it. Such antifungal properties did not remain limited to the planktonic form of *C. albicans*, as the eradication of biofilms upon exposure to **132** was confirmed in vitro by examining biomass and CFU reduction. Additionally, **131** and **132** were found to be suitable for in vivo testing, due to their negligible rate of hemolysis in murine red blood and their lack of toxicity toward 293T cells. However, infected wounds on mice subsequently treated with **132** underwent visually (Figure 14A) and quantitatively (Figure 14B,C) faster healing process by comparison with administration of ZIF-8 or even Vrz [51].

Stevanovic et al. [74] produced a previously discussed publication in which they assessed the improvement of drug potency upon complexation on an Au(III) metallic core. Among the investigated complexes, the Vrz-containing compound **133** (Scheme 2) was particularly addressed by the authors question, as it yielded the highest general increase in antifungal activity with respect to the corresponding uncoordinated AA. Indeed, an almost 30-fold reduction of MIC value was measured against *C. auris*, for instance (Table 4). However, because Vrz was by far the less efficient AA considered in this work, these promising data have to be received carefully. Furthermore, **133** showed higher MIC values against all tested bacterial strains by comparison with the other AA complexes **25**, **26** and **29**, although it still improved the antibacterial capacity of free Vrz by a factor ranging from 1.55 to 7.47 (Table 2) [74].

## 3. Conclusions

The versatility of AAs as therapeutical agents and their ability to coordinate to metal cores have enabled the discovery of powerful and medically relevant compounds. These coordination species could often yield better antimicrobial or anticancer activity than the free AA and, in some cases, even overcome resistance with a distinct mechanism of action. Such an observable MDS constitutes an encouraging outcome in the fight against AMR, NTDs and the rise of cancer incidence.

Nevertheless, the potential of these promising molecules has not yet been fully exploited. Indeed, few research groups have been exploring this field, which resulted in high structural similarity among the reported complexes. For example, Cu, Zn or Ru, and more particularly its piano stool scaffolds bearing a Ctz ligand, have been discussed in a considerable number of publications cited above, whereas other usually common metals, like Fe, Co or Pd, have barely been mentioned. Furthermore, most AAs have scarcely or never been complexed to a metal center for medical purposes. One could think of Bfz, which was only discussed once in this document, as well as isoconazole or terconazole, which were not investigated in a single study reported here.

In summary, the complexation of AAs for the finding of new potent antibiotics and cytotoxic molecules constitutes a promising, but relatively young research area. Therefore, it still presents important opportunities for great discoveries.

## Figures and Tables

**Table 1:** Anticancer properties of selected complexes featuring one or more AA ligands.

Complex	Cancerous Cell Line	IC_50_ Value (µM) wAA	IC_50_ Value (µM) of the AA (Ratio)	IC_50_ Value (µM) of Cisplatin (Ratio)	IC_50_ Value (µM) against Healthy Cell Lines (SI)							References
**3**	HCT15	6.78	>516 (>71.1)	10.46 (1.543)								[54]
**6**	HeLa	27.067	>516 (>19.1)	200.8 (7.419)								[54]
**9**	HeLa	15.14	>258 (>17.0)	18.43 (1.217)								[55]
**18**	MCF7	1.9	12.5 ^b^ (6.6)									[56]
	MCF7 ^a^	1.4	15.3 ^b^ (11)									
	LNCaP	4.2	8.6 ^b^ (2.0)									
	LNCaPa	0.4	10.4 ^b^ (30)									
	PC3	13.6	18.4 ^b^ (1.35)									
	PC3a	1.8	15.1 ^b^ (8.4)									
	DLD1	8.1	42.5 ^b^ (5.2)									
	DLD1 ^a^	3.2	43.4 ^b^ (14)									
**19**	MCF7	20.8	12.5 ^b^ (0.601)									[56]
	MCF7 ^a^	2	15.3 ^b^ (8)									
	LNCaP	18.5	8.6 ^b^ (0.46)									
	LNCaP ^a^	0.6	10.4 ^b^ (20)									
	PC3	98.4	18.4 ^b^ (0.187)									
	PC3 ^a^	5.2	15.1 ^b^ (2.9)									
	DLD1	57.2	42.5 ^b^ (0.743)									
	DLD1a	5.70	43.4 ^b^ (7.61)									
**20**	HepG2	10.5		25.5 ^c^ (2.43)								[57]
	MCF7	5.96		19.0 ^d^ (3.19)								
**21**					Balb/c3T3							[58]
	HepG2	0.92	>1 (>1)	>1 (>1)	1.1 (1.2)							
		0.28 ^e^	>1 (>4)	<0.0001 (<0.0004)	0.50 (1.8)							
**22**					Balb/c3T3							[59]
	HepG2	0.90	>1 (>1)	>1 (>1)	1.1 (1.2)							
		0.24 ^e^	>1 (>4)	<0.0001 (<0.0004)	0.48 (2.0)							
**37**					HaCaT			MCF10A		MEFs		[60]
	HeLa	36.9 ^f^	94.3 (2.56)		26.1		(0.707)	42.9	(1.16)	5.6	(0.15)	
	LNCaP	5.3 ^f^	15.4 (2.9)				(4.9)		(8.1)		(1.1)	
	YT	5.2 ^f^	16.7 (3.2)				(5.0)		(8.2)		(1.1)	
**41**	A2780	0.54	5.7 (11)	1.9 (3.5)								[61]
	HeLa	3.6	18.4 (5.1)	7.00 (1.9)								
	MCF7	5.4	20.5 (3.8)	28 (5.2)								
**42**					L929				MRC5			[62]
	A549	0.61	14.47 (24)	14.42 (24)	1.15	(1.9)			1.16		(1.9)	
	DU145	5.13	15.82 (3.08)	2.33 (0.454)		(0.224)					(0.226)	
	MDAMB231	0.63	10.11 (16)	2.44 (3.9)		(1.8)					(1.8)	
**43**					HaCaT			MCF10A		MEFs		[60]
	22Rv1	7.2 ^f^	19.2 (2.7)		NA (not active)			39.0	(5.4)	9.6	(1.3)	
	HeLa	76.4 ^f^	94.3 (1.23)						(0.510)		(0.13)	
	LNCaP	4.8 ^f^	15.4 (3.2)						(8.1)		(2.0)	
	YT	7.2 ^f^	16.7 (2.3)						(5.4)		(1.3)	
**44**					HaCaT			MCF10A		MEFs		[60]
	LNCaP	10 ^f^	15.4 (1.5)		NA			67.0	(6.7)	9.0	(0.90)	
	YT	8.6 ^f^	16.7 (1.9)						(7.8)		(1.0)	
**53**	HCT15	13	52.3 (4.0)	4.6 (0.35)								[63]
	HeLa	7.8	12.4 (1.6)	5.2 (0.67)								
	PC3	9.8	12.3 (1.3)	19.0 (1.9)								
**54**	HCT15	14.4	52.3 (3.63)	4.6 (0.32)								[63]
	HeLa	10.5	12.4 (1.18)	5.2 (0.50)								
**71**					L929							[64]
	A549	2.11	14.47 (6.86)	14.42 (6.83)	2.61				(1.24)			
	DU145	3.64	15.82 (4.35)	2.33 (0.640)					(0.717)			
	MCF7	3.83	16.80 (4.39)	13.98 (3.65)					(0.681)			
**72**					L929							[64]
	A549	0.47	14.47 (31)	14.42 (31)	0.48				(1.0)			
	DU145	3.32	15.82 (4.77)	2.33 (0.702)					(0.14)			
	MCF7	1.68	16.80 (10.0)	13.98 (8.32)					(0.29)			
**73**					L929							[64]
	A549	4.79	14.47 (3.02)	14.42 (3.01)	5.89				(1.23)			
	DU145	7.40	15.82 (2.14)	2.33 (0.315)					(0.796)			
	MCF7	13.28	16.80 (1.265)	13.98 (1.053)					(0.444)			
**77**					ARPE19							[65]
	BE	8.5		0.7 (0.08)	32.5				(3.8)			
	MiaPaCa2	4.9		2.8 (0.57)					(6.6)			
	Panc10.05	1.9		1.7 (0.89)					(17)			
**81**					ARPE19							[65]
	BE	3.6		0.7 (0.2)	8.7 (2.4)							
**82**					ARPE19							[65]
	BE	18.0		0.7 (0.04)	>50				(>2.8)			
	MiaPaCa2	11.7		2.8 (0.24)					(>4.3)			
	Panc10.05	7.1		1.7 (0.24)					(>7.0)			
**83**					ARPE19							[65]
	BE	9.0		0.7 (0.08)	41.2				(4.6)			
	MiaPaCa2	8.6		2.8 (0.33)					(4.8)			
	Panc10.05	6.5		1.7 (0.26)					(6.3)			
**84**					ARPE19				BHK21			[65,66]
	BE	3.3		0.7 (0.2)	24.2	(7.3)			23.0			
	B16F1	19.3	48 ^g^ (2.5)	5.4 (0.28)							(1.2)	
	CT26WT	12.2	46.2 ^g^ (3.79)	6.0 (0.49)							(1.9)	
**85**					ARPE19							[65]
	BE	3.1		0.7 (0.2)	26.0 (8.4)							
**86**					BHK21							[66]
	B16F1	14	48 ^g^ (3.4)	5.4 (0.39)	37.6				(2.7)			
	CT26WT	15	46.2 ^g^ (3.1)	6.0 (0.40)					(2.5)			
**87**	HepG2	8.27		25.5 ^c^ (3.08)								[57]
	MCF7	6.97		19.0 ^d^ (2.73)								
**89**	A2780	6.1		10.5 (1.7)								[67]
	A549	19.0		38.8 (2.04)								
	SW480	38.3		45.1 (1.18)								
**94**					HaCaT			MCF10A		MEFs		[60]
	22Rv1	21.4 ^f^	24.2 (1.13)		NA			6.7	(0.31)	5.2	(0.24)	
	DU145	8.1 ^f^	28.6 (3.5)						(0.83)		(0.64)	
	HeLa	47.8 ^f^	66.7 (1.40)						(0.14)		(0.11)	
	LNCaP	4.3 ^f^	27.9 (6.5)						(1.6)		(1.2)	
**95**					HaCaT			MCF10A		MEFs		[60]
	22Rv1	7.4 ^f^	24.2 (3.3)		61.6		(8.3)	66.4	(9.0)	25.3	(3.4)	
	HeLa	61.4 ^f^	66.7 (1.09)				(1.00)		(1.08)		(0.412)	
**96**					HaCaT			MCF10A		MEFs		[60]
	22Rv1	14.7 ^f^	24.2 (1.65)		312.6		(21.3)	171	(11.6)	39.2	(2.67)	
	LNCaP	6.6 ^f^	27.9 (4.2)				(47)		(26)		(5.9)	
**97**					HaCaT			MCF10A		MEFs		[60]
	HeLa	33.3 ^f^	66.7 (2.00)		113.1		(3.40)	12.3	(0.369)	37.3	(1.12)	
	LNCaP	6.8 ^f^	27.9 (4.1)				(17)		(1.8)		(5.5)	
**98**					HaCaT			MCF10A		MEFs		[60]
	LNCaP	7.4 ^f^	27.9 (3.8)		NA			NA		212.5	(29)	
	YT	<4 ^f^	4.9 (>1)								(>50)	
**99**					L929				MRC5			[60]
	A549	0.64	41.85 (65)	14.42 (23)	1.09	(1.7)			1.80		(2.8)	
	DU145	4.45	47.54 (10.7)	2.33 (0.524)		(0.245)					(0.404)	
	MDAMB231	0.62	10.26 (17)	2.44 (3.9)		(1.8)					(2.9)	
**110**					BHK21							[66]
	B16F1	2.8	4.3 ^g^ (1.5)	5.4 (1.9)	26.3				(9.4)			
	CT26WT	16.1	45.4 ^g^ (2.82)	6.0 (0.37)					(1.63)			
**111**					BHK21							[66]
	B16F1	4.4	4.3 ^g^ (0.98)	5.4 (1.2)	48.4				(11)			
	CT26WT	31.4	45.4 ^g^ (1.45)	6.0 (0.19)					(1.54)			
**123**	KB31	26.08	>100 (>3.834)									[68]
	SNB19	55.60	>100 (>1.799)									
**129**					L929				MRC5			[62]
	A549	2.94	>100 (>34.0)	14.42 (4.90)	2.00	(0.680)			2.02		(0.687)	
	DU145	3.90	>100 (>25.6)	2.33 (0.597)		(0.513)					(0.518)	
	MDAMB231	2.35	>100 (>42.6)	2.44 (1.04)		(0.851)					(0.860)	

^a^ These cell lines were exposed to sunlight while treated with the complex. ^b^ These values correspond to measurements performed with the AA nitrate salt. ^c^ The authors reported this value from another work [69]. ^d^ The authors reported this value from another work [70]. ^e^ These data were reported as the corresponding IC_20_ value. ^f^ These data were reported as the corresponding CC_50_ value. ^g^ These values correspond to measurements performed with an NHC derivative of the AA.

**Table 2:** Antibacterial properties of selected complexes featuring one or more AA ligands.

Complex	Bacterial Pathogen		MIC Value (µM) wAA	MIC Value (µM) of the AA (Ratio)	Control Salt	MIC Value (µM) of the Control Salt (Ratio ^a^)	References
	G+	G−					
**16**		*E. coli*	20	>40 (>2.0)			[73]
	*E. faecium*		1.25	10 (8.0)			
	*E. faekalis*		2.5	10 (4.0)			
	*S. aureus*		1.25	5 (4)			
	*S. epidermis*		1.25	2.5 (2.0)			
		*Y. pestis*	10	>40 (>4.0)			
		*Y. pseudotuberculosa*	20	>40 (>2.0)			
**21**	*M. luteus*		0.98	1.18 (1.2)	AgNO_3_	11.40 (23)	[59]
**22**		*P. mirabilis*	120.4	>1000 (>8.306)	AgClO_4_	151.21 (2.512)	[59]
**25**		*E. coli*	36.5	>450 (>12.3)	[Au(imz)Cl_3_]	67.5 (1.85)	[74]
		*P. aeruginosa* (PA14)	18.3	>450 (24.6)		67.5 (3.69)	
	*S. aureus* (ATCC 259233)		18.3	225 (12.3)		67.5 (3.69)	
	*S. aureus* (MRSA)		9.1	14.1 (1.5)		67.5 (7.4)	
**26**		*E. coli*	36.2	516 (14.3)	[Au(imz)Cl_3_]	67.5 (1.86)	[74]
		*P. aeruginosa* (PA14)	18.1	516 (28.6)		67.5 (3.73)	
	*S. aureus* (ATCC 259233)		72.4	258 (3.56)		67.5 (0.932)	
	*S. aureus* (MRSA)		18.1	32.2 (1.78)		67.5 (3.73)	
**29**		*E. coli*	30.9	>580 (>18.8)	[Au(imz)Cl_3_]	67.5 (2.18)	[74]
		*P. aeruginosa* (PA14)	38.6	>580 (>15.0)		67.5 (1.75)	
	*S. aureus* (ATCC 259233)		19.3	290 (15.0)		67.5 (3.50)	
	*S. aureus* (MRSA)		19.3	36.3 (1.88)		67.5 (3.50)	
**55**	*E. faecium*		2.5	20 (8.0)			[73]
	*E. faekalis*		2.5	40 (16)			
	*S. aureus*		0.625	20 (32)			
	*S. epidermis*		0.625	2.5 (4.0)			
**56**	*S. aureus*		3	10 (3)			[75]
**57**		*A. baumannii*	9	>93 (>10)	[Re(CO)_3_(bpy)Br]	>63 (>7)	[75,76]
	*B. subtilis*		0.27	6 (20)		>63 (>230)	
		*E. aerogenes*	35	>93 (>2.7)		>63 (>1.8)	
		*E. coli*	17	>93 (>5.5)		>63 (>3.7)	
	*E. faecium*		2	20 (10)		>63 (>30)	
		*K. pneumoniae*	35	>93 (>2.7)		>63 (>1.8)	
		*P. aeruginosa*	35	>93 (>2.7)		>63 (>1.8)	
	*S. aureus* (ATCC 29213)		0.27	20 (74)		>63 (>230)	
	*S. aureus* (NCTC 8325)		0.27	10 (37)		>63 (>230)	
	*S. aureus* (MRSA)		0.5	10 (20)		>63 (>100)	
	*S. aureus*		4				
			9 ^b^				
	*S. aureus* (MRSA)		4				
			9 ^b^				
**58**	*S. aureus*		0.6	10 (20)			[75]
**59**	*S. aureus*		0.6	10 (20)			[75]
**66**	*S. aureus*		8				[76]
			>8 ^b^				
	*S. aureus* (MRSA)		8				
			>8 ^b^				
**67**	*S. aureus*		8				[76]
			>8 ^b^				
	*S. aureus* (MRSA)		8				
			>8 ^b^				
**68**	*S. aureus*		7				[76]
			7 ^b^				
	*S. aureus* (MRSA)		7				
			7 ^b^				
**70**	*S. aureus*		22.7	90.62 (3.99)			[77]
	*S. aureus* (MRSA)		36.34	90.62 (2.494)			
**70 ^c^**	*S. aureus*		7.3	302 (41)			[77]
	*S. aureus* (MRSA)		7.3	302 (41)			
**71**	*M. tuberculosis*		10.52	70.25 (6.678)			[64]
**72**	*M. tuberculosis*		10.30	70.25 (6.820)			[64]
**73**	*M. tuberculosis*		18.53	70.25 (3.791)			[64]
**100**	*E. faecium*		10	>40 (>4.0)			[73]
	*E. faekalis*		20	>40 (>2.0)			
	*S. aureus*		2.5	40 (16)			
	*S. epidermis*		2.5	40 (16)			
**133**		*E. coli*	76.6	572 (7.47)	[Au(imz)Cl_3_]	67.5 (0.881)	[74]
		*P. aeruginosa* (PA14)	76.6	572 (7.47)		67.5 (0.881)	
	*S. aureus* (ATCC 259233)		153	572 (3.74)		67.5 (0.441)	
	*S. aureus* (MRSA)		76.6	572 (7.47)		67.5 (0.881)	

^a^ This value was multiplied by the number of AA ligands in the corresponding complex in order to compare the activity of this complex with that of its control salt. ^b^ These data were reported as the corresponding MBC values. ^c^ This complex was encapsulated in solid lipid nanoparticles.

**Table 3:** Antikinetoplastid properties of selected complexes featuring one or more AA ligands.

Complex	Kinetoplastid	IC_50_ Value (µM) wAA	IC_50_ Value (µM) of the AA (Ratio)	IC_50_ Value (µM) against Healthy Cell Lines (SI)							References
**16**				293T				J774.1			[73]
	*L. major*	1.8	42.6 (24)	1.9		(1.1)		1.8		(1.0)	
	*T. brucei*	0.4	17.5 (40)			(4.8)				(4.5)	
**27**				Vero							[78]
	*T. cruzi* ^a^	0.2 ^b^	3 (10)	NA (not active)							[79]
	*T. cruzi* ^c^	0.01 ^b^	0.01 (1)								
**37**				Human osteoblast							[80]
	*L. major* ^d^	0.015 ^e^	1.6 (110)	>7.5				(>500)			
	*L. major* ^c^	0.029 ^f^	>1 (>30)								
	*T. cruzi* ^a^	0.1 ^e^	5.8 (60)					(>70)			
**40**				Human osteoblast							[80]
	*L. major* ^d^	0.45 ^e^	1.6 (3.6)	6.55				(15)			
	*T. cruzi* ^a^	2.9 ^e^	5.8 (2.0)					(2.3)			
**41**				Murine macrophages							[61]
	*T. brucei*	0.6	>25 (>40)	1.9				(3)			
	*T. cruzi* ^a^	0.25	1.8 (7.2)					(7.6)			
**42**	*L. amazonensis* ^d^	0.57	0.87 (1.5)								[81]
	*L. amazonensis* ^c^	0.02580									
**55**				293T				J774.1			[73]
	*L. major*	2.2	64.6 (29)	<1.25		(<0.57)		1.9		(0.86)	
	*T. brucei*	0.5	17.4 (30)			(<2)				(4)	
**61**				Vero							[82,83]
	*T. cruzi* ^a^	2.43		5.10							
	*T. cruzi* ^a^	3.48	22.2 (6.38)					(1.47)			
	*T. cruzi* ^g^	0.61	10.2 (17)					(8.4)			
**62**				Vero							[83]
	*T. cruzi* ^a^	9.42	22.2 (2.36)	3.20				(0.340)			
	*T. cruzi* ^g^	1.28	10.2 (7.97)					(2.50)			
**63**				Vero							[83]
	*T. cruzi* ^a^	7.53	22.2 (2.95)	6.50				(0.863)			
	*T. cruzi* ^g^	1.11	10.2 (9.19)					(5.86)			
**64**				Vero							[83]
	*T. cruzi* ^a^	8.48	22.2 (2.62)	14.0				(1.65)			
	*T. cruzi* ^g^	2.26	10.2 (4.51)					(6.19)			
**65**				Vero							[83]
	*T. cruzi* ^a^	8.43	22.2 (2.63)	12.8				(1.52)			
	*T. cruzi* ^g^	2.79	10.2 (3.66)					(4.59)			
**74**				HFF							[84]
	*T. vaginalis*	9.8	17.2 (1.8)	56.8 (5.8)							
**94**				Hs27		IPφ		RAW264.7		U2OS	[85]
	*L. major* ^d^	0.8 ^e^	1.9 (2)	>120	(>100)	>3.5	(>4)	>7.5	(>9)	120	(100)
	*T. cruzi* ^a^	1.39 ^e^	1.5 (1.1)		(>86.3)		(>2.5)		(>5.4)		(86.3)
**99**	*L. amazonensis* ^d^	0.15	>3.00 (>20)								[81]
	*L. amazonensis* ^c^	0.01553									
**100**				293T				J774.1			[73]
	*L. major*	2.0	66.0 (33)	6.3		(3.1)		8.2		(4.1)	
	*T. brucei*	0.7	20.5 (30)			(10)				(10)	
**103**				Raw264.7		LLCMK2		HFF			[86]
	*L. amazonensis* ^d^	0.008	0.044 (5)								
	*L. amazonensis* ^c^	0.000336	0.14538 (433)	1.60 (4760)							
	*T. cruzi* ^a^	0.00662	0.0870 (13.1)								
	*T. cruzi* ^c^	0.00228	0.0023 (1.0)			0.73782 (324)					
	*T. gondii*	0.0744	0.1623 (2.18)					>10.00 (>134)			
**104**				Raw264.7		LLCMK2		HFF			[86]
	*L. amazonensis* ^d^	0.0032	0.044 (14)								
	*L. amazonensis* ^c^	0.06614	0.14538 (2.198)	1.20 (18.1)							
	*T. cruzi* ^a^	0.0026	0.0870 (33)								
	*T. cruzi* ^c^	0.0018	0.0023 (1.3)			1.011 (560)					
	*T. gondii*	0.120	0.1623 (1.35)					>10.00 (>83.3)			
**123**				HFFL							[87]
	*T. cruzi* ^a^	3.56	10.6 (2.98)	107				(30.1)			
	*T. cruzi* ^c^	12	24.3 (2.0)					(8.9)			
**124**				HFFL							[87]
	*T. cruzi* ^a^	1.7	10.6 (6.2)	860				(510)			
	*T. cruzi* ^c^	10	24.3 (2.4)					(86)			
	*T. cruzi* ^g^	6.30	7.04 (1.12)					(137)			
**125**				HFFL							[87]
	*T. cruzi* ^a^	1.9	10.6 (5.6)	171				(90)			
	*T. cruzi* ^c^	13	24.3 (1.9)					(13)			
**127**				HFFL							[87]
	*T. cruzi* ^a^	2.54	10.6 (4.17)	760				(299)			
	*T. cruzi* ^c^	13	24.3 (1.9)					(58)			
**128**				HFFL							[87]
	*T. cruzi* ^a^	3.0	10.6 (3.5)	>1000				(>330)			
	*T. cruzi* ^c^	9.90	24.3 (2.45)					(>101)			

^a^ The epimastigote form of these parasites was used during the tests. ^b^ These data were reported as the corresponding EC_50_ value. ^c^ The amastigote form of these parasites was used during the tests. ^d^ The promastigote form of these parasites was used during the tests. ^e^ These data were reported as the corresponding LD_50_ value. ^f^ These data were reported as the corresponding IC_70_ value. ^g^ The trypomastigote form of these parasites was used during the tests.

**Table 4:** Antifungal properties of selected complexes featuring one or more AA ligands.

Complex	Fungus	MIC Value (µM) wAA	MIC Value (µM) of the AA (Ratio)	Control Salt	MIC Value (µM) of the Control Salt (Ratio ^a^)	References
**21**	*C. albicans*	2.0	4.69 (2.3)	AgNO_3_	11.40 (11)	[59]
	*C. glabrata*	3.90	4.69 (1.20)		11.40 (5.85)	
**22**	*C. albicans*	3.76	4.69 (1.25)	AgClO_4_	9.42 (5.01)	[59]
	*C. glabrata*	3.76	4.69 (1.25)		9.42 (5.01)	
**23**	*C. albicans*	3.80	4.69 (1.23)	AgBF_4_	20.10 (10.6)	[59]
	*C. glabrata*	3.80	4.69 (1.23)		10.05 (5.29)	
**24**	*C. albicans*	3.32	4.69 (1.41)	AgSbF_6_	11.37 (6.85)	[59]
	*C. glabrata*	1.7	4.69 (2.8)		11.37 (13)	
**25**	*C. albicans*	4.6	7.0 (1.5)	[Au(imz)Cl_3_]	135 (29)	[74]
	*C. glabrata*	9.1	56.2 (6.2)		135 (15)	
	*C. parapsilosis*	2.5	3.9 (1.6)		135 (54)	
**26**	*C. albicans*	0.6	2.3 (4)	[Au(imz)Cl_3_]	135 (200)	[74]
	*C. auris*	3.6	8.1 (2.2)		270 (75)	
	*C. glabrata*	9.0	64.5 (7.2)		135 (15)	
	*C. krusei*	1.5	32.2 (21)		67.5 (45)	
**29**	*C. auris*	1.9	9.1 (4.8)	[Au(imz)Cl_3_]	270 (140)	[74]
	*C. glabrata*	4.8	9.1 (1.9)		135 (28)	
	*C. krusei*	0.4	1.4 (3)		67.5 (200)	
	*C. parapsilosis*	1.4	10.2 (7.3)		135 (96)	
**35**	*S. schenckii*	0.001	0.005 (5)	[Au(PPh_3_)Cl]	0.002 (2)	[89]
		0.001 ^b^	0.020 (20)		0.008 (8)	
**51**	*C. albicans*	0.1 ^c^	0.5 (5)			[90]
	*C. neoformans*	0.250 ^c^	0.5 (2)			
	*S. brasiliensis*	0.250 ^c^	0.5 (2)			
**74**	*C. albicans*	0.50 ^c^	0.5 (1)			[90]
	*C. neoformans*	0.50 ^c^	0.5 (1)			
	*S. brasiliensis*	0.06 ^c^	0.5 (8)			
**76**	*C. albicans*	0.250 ^c^	0.5 (2)			[90]
	*C. neoformans*	0.50 ^c^	0.5 (1)			
	*S. brasiliensis*	0.06 ^c^	0.5 (8)			
**92**	*S. schenckii*	0.0002	0.0009 (4)	[Au(PPh_3_)Cl]	0.002 (10)	[89]
		0.001 ^b^	>0.040 (>40)		0.008 (8)	
**93**	*S. schenckii*	0.004	0.0009 (0.2)	[Cu(PPh_3_)_2_(NO_3_)]	>0.02 (>10)	[89]
		0.008 ^b^	>0.040 (>5)		>0.02 (>5)	
**103**	*S. brasiliensis*	0.2	0.6 (3)	ZnCl_2_	>20 (>200)	[86]
	*S. schenckii*	0.2	1.25 (6)		>20 (>200)	
		5.0 ^b^	>20 (>4.0)		>20 (>8.0)	
**104**	*S. brasiliensis*	0.6	0.6 (1)	Zn(ac)_2_	>20 (>70)	[86]
	*S. schenckii*	0.32	1.25 (3.9)		>20 (>120)	
**105**	*C. albicans*	0.38	0.44 (1.2)			[91]
	*C. glabrata*	0.78	1.77 (2.3)			
	*C. parapsilosis*	0.38	0.44 (1.2)			
**106**	*C. albicans* ^d^	0.39	3.13 ^f^ (8.0)			[92]
	*C. albicans* ^e^	0.78	50 ^f^ (64)			
**107**	*C. albicans* ^d^	0.39	3.13 ^f^ (8.0)			[92]
	*C. albicans* ^e^	0.78	50 ^f^ (64)			
**108**	*C. albicans*	0.1 ^c^	0.25 (2)			[90]
	*C. neoformans*	<0.008 ^c^	0.06 (>7)			
	*S. brasiliensis*	0.030 ^c^	0.03 (1)			
**109**	*C. albicans*	0.06 ^c^	0.25 (4)			[90]
	*C. neoformans*	<0.008 ^c^	0.06 (>7)			
	*S. brasiliensis*	0.030 ^c^	0.03 (1)			
**112**	*A. niger*	10 ^g^	>2090 (>210)			[93]
	*C. albicans*	0.53 ^g^	0.82 (1.5)			
	*M. mucedo*	10 ^g^	65 (6.5)			
	*P. uniculosum*	21 ^g^	2090 (100)			
	*R. tolonifer*	10 ^g^	65 (6.5)			
	*S. cerevisiae*	4 ^g^	>210 (>50)			
**113**	*A. niger*	24 ^g^	>2090 (>87)			[93]
	*C. albicans*	0.61 ^g^	0.82 (1.3)			
	*M. mucedo*	10 ^g^	65 (6.5)			
	*P. uniculosum*	24 ^g^	2090 (87)			
	*R. tolonifer*	10 ^g^	65 (6.5)			
	*S. cerevisiae*	5 ^g^	>210 (>40)			
**114**	*C. albicans*	2 ^i^	46 (20)	Ag(ClO_4_)	10 (10)	[50]
	*C. albicans* ^h^	4 ^i^	20 (5)		10 (5)	
	*C. albicans* ^h^	4 ^i^	>180 (>40)		5 (2)	
	*C. albicans* ^h^	4 ^i^	46 (10)		10 (5)	
	*C. albicans* ^h^	4 ^i^	180 (40)		10 (5)	
**115**	C. albicans	1 ^i^	46 (50)	Ag(NO_3_)	10 (20)	[50]
	*C. albicans* ^h^	2 ^i^	20 (10)		6 (6)	
	*C. albicans* ^h^	2 ^i^	>180 (>90)		3 (3)	
	*C. albicans* ^h^	2 ^i^	46 (20)		6 (6)	
	*C. albicans* ^h^	1 ^i^	180 (200)		3 (6)	
**118**	*A. flavus*	0.1	0.6 (6)			[94]
	*A. niger*	0.1	0.6 (6)			
**119**	*A. flavus*	0.1	0.6 (6)			[94]
	*A. niger*	0.1	0.6 (6)			
	*C. neoformans*	0.1	0.6 (6)			
**122**	*C. albicans* (HL 3968)	2 ^c^	10 (5)			[95]
		20 ^g^	100 (5)			
	*C. albicans* (HL 973)	4 ^c^	52 (10)			
		8 ^g^	210 (30)			
**124**	*C. krusei*	7.42	40.81 (5.50)			[96]
	*C. parapsilosis*	2.09	5.71 (2.73)			
**131**	*C. albicans*	0.23	0.29 (1.3)	ZIF-8	NA (not active)	[51]
		0.91 ^b^	2.2 (2.4)			
	*C. albicans* ^j^	0.029	0.29 (10)			
		0.11 ^b^	2.2 (20)			
**132**	*C. albicans*	0.52	0.29 (0.56)	ZIF-8	NA	[51]
		2.1 ^b^	2.2 (1.0)			
	*C. albicans* ^j^	0.066	0.29 (4.4)			
		0.26 ^b^	2.2 (8.5)			
**133**	*C. albicans*	4.8	35.8 (7.5)	[Au(imz)Cl_3_]	135 (28)	[74]
	*C. auris*	19.1	572 (29.9)		270 (14.1)	
	*C. glabrata*	76.6	572 (7.47)		135 (1.76)	

^a^ This value was multiplied by the number of AA ligands in the corresponding complex in order to compare the activity of this complex with that of its control salt. ^b^ These data were reported as the corresponding MFC value. ^c^ These data were reported as the corresponding MIC_50_ value. ^d^ These strains were Fcz-sensitive clinical isolates. ^e^ These strains were Fcz-resistant clinical isolates. ^f^ These values correspond to measurements performed with a phosphine derivative of Ktz (KtzP). ^g^ These data were reported as the corresponding MIC_80_ value. ^h^ This strain was a Vrz-resistant clinical isolate. ^i^ These data were reported as the corresponding MIC_90_ value. ^j^ Upon treatment of these fungi with the coordination compound, the pH of the medium was adjusted at 5.0.
